# Humans: Intertwined with life and the basic elements in the Anthropocene biosphere

**DOI:** 10.1007/s13280-026-02474-z

**Published:** 2026-09-04

**Authors:** Carl Folke, Andrew Hattle, Peter Søgaard Jørgensen, Henrik Österblom, Tone Bjordam, Gregory Bratman, Connor Lashus, Staffan Normark, Lan Wang-Erlandsson, Gretchen Daily, Jonathan Donges, Eric Galbraith, Esteban Jobbágy, Ariane König, Tomás Milani, Magnus Nyström, Belinda Reyers, Juan Rocha, Johan Rockström, Caroline Schill

**Affiliations:** 1 The Anthropocene Laboratory, The Royal Swedish Academy of Sciences, Box 50005, 104 05 Stockholm, Sweden; 2 Nordmarka, Oslo, Norway; 3 School of Environmental and Forest Sciences, University of Washington, Anderson Hall, Box 352100, Seattle, WA 98195-2100 USA; 4 Karolinska Institutet, 171 77 Stockholm, Sweden; 5 Stockholm Resilience Centre, Stockholms Universitet, Frescativägen 8, 106 91 Stockholm, Sweden; 6 NatCap at Stanford, 327 Campus Drive, Bass Biology Building 123, Stanford, CA 94305 USA; 7 Potsdam Institute for Climate Impact Research (PIK), P.O. Box 60 12 03, 14412 Potsdam, Germany; 8 ICREA, Passeig Lluís Companys, 23, 08010 Barcelona, Spain; 9 CCT CONICET San Luis, Almirante Brown 907, D5700HHW, San Luis, Argentina; 10 Department of Social Sciences, University of Luxembourg, 11, porte des Sciences, 4366 Esch-sur-Alzette, Luxembourg; 11 Centre for Environmental Studies, University of Pretoria, Private Bag X20 Hatfield, Pretoria, 0028 South Africa

**Keywords:** Anthropocene, Biosphere, Intertwined, Metaorganism, Social-ecological systems, Stewardship

## Abstract

The notion of people and nature as intertwined and embedded in the biosphere is gaining traction in contemporary scientific discourses. But what does it mean for a human being to be intertwined? Here, we explore this question through two lenses: looking at the human body as an open living system in continuous exchange with the chemical elements on Earth; and looking across the biosphere, at the intricate functioning of the web of life in mediating these exchanges. The first lens focuses on relationships between chemical elements and life processes within us humans, relationships that are critical for our bodies to operate and function. We then expand the perspective to show how microorganisms, fungi, plants, and animals in ecosystems shape and mediate the elemental flows through the air, water, and food that our bodies require for survival. The two lenses highlight that humans have evolved as part of, and remain integrated with, these complex and dynamic life–element interdependencies, spanning all scales and domains across the biosphere. In the Anthropocene, humans remain as bodily dependent on life-mediated elemental cycles as ever, irrespective of culture, economic development, or technological advances. This inseparability reflects the core reality of existing in the biosphere, with implications for how to approach and understand human–environment relations and confront the challenges of the Anthropocene. It further implies that biosphere stewardship is not only a cognitive or ethical orientation, but also an embodied practice: one that involves learning about, caring for, and enhancing the living relations that mobilise, regenerate, and circulate life-sustaining elements.

**Supplementary Information:**

The online version contains supplementary material available at https://doi.org/10.1007/s13280-026-02474-z.

*Omnia mirari etiam tritissima* (Marvel at everything, even the most mundane) Carl Linnaeus

## Introduction

In the Anthropocene, the polycrisis, including climate change (Delannoy et al. 2025) and the overshoot of planetary boundaries (Richardson et al. 2023), underscores that the future of civilisations rests on an Earth system that is liveable for humankind (Steffen et al. 2018). Collaboration and active stewardship of the human-nature relationship are necessary (Guerry et al. 2015; Chapin et al. 2022). Humans and nature are not only linked and interacting; they are deeply intertwined as part of the living planet (Folke et al. 2011; Tsing 2015; Haraway 2016; Schlüter et al. 2020), an appreciation that has long been central to many Indigenous and relational knowledge systems (Johannes 1981; Berkes 1999; West et al. 2020; Gould et al. 2023).

People exist in a unique thin layer—the biosphere—a ‘critical zone of life’ extending roughly 20 km vertically, with most of the action occurring in the upper 200 m of the ocean and on land in a surface layer just tens of metres thick. This is where life has emerged and evolved, human life included (Wilson 1992). Irrespective of gender, political system, religion, culture, all humans are embedded in the biosphere (Folke et al. 2016; Latour and Weibel 2020; Dasgupta 2021).

**Fig. 1:**
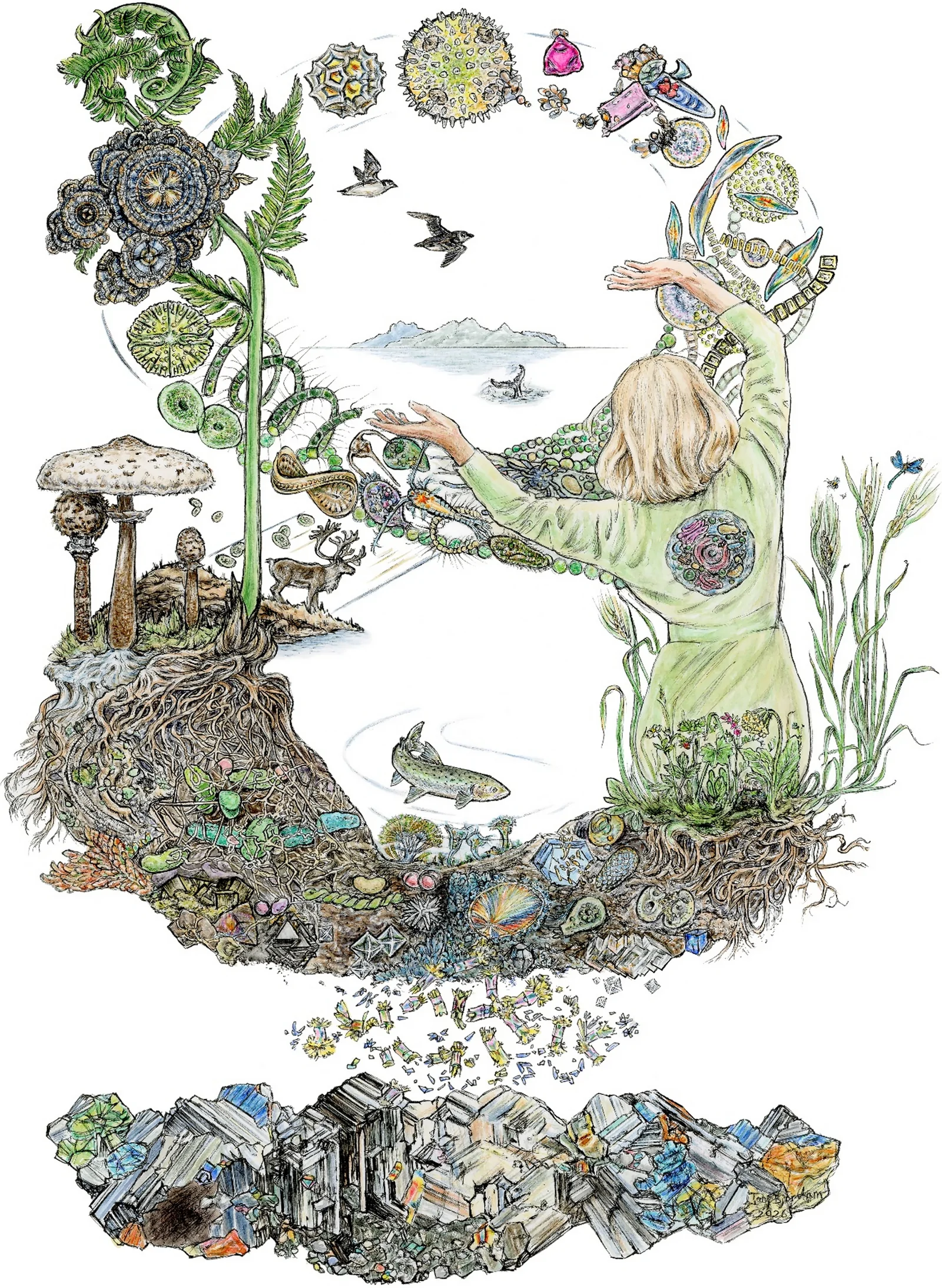
*The Elements of Life*, ink drawing and aquarelle on watercolour paper, Tone Bjordam, 2026: an interpretive synthesis of life-mediated elemental flows illustrating that *humans are intertwined beings embodying the biosphere*. The cyclic composition situates a human figure within, rather than outside, the biosphere. The bottommost elemental, crystalline and mineral (inspired by cordierite) forms represent the geochemical foundations of life, while a human cell and surrounding microorganisms (an alphaproteobacterium; an Asgard archaeon, *Candidatus Lokiarchaeum ossiferum*; a deltaproteobacterium*;* and a paramecium, *Paramecium* sp.) connect elemental processes within the human body to those operating through soil, water and air. Reflecting the mobilisation, transformation, transport, incorporation and recycling of elements through living systems, the figure interweaves roots, mycelia, soil organisms (a nematode; soil bacteria including manganese-oxidising bacteria; and testate amoebae including *Euglypha tuberculata* and *Arcella* sp.), and elemental, mineral and crystalline forms distributed through the surrounding composition and soil (crystalline representations of copper, sulphur, calcium and magnesium; and compounds including copper oxide, sodium chloride, calcium carbonate, magnesium sulphate, zinc oxide, chalcopyrite, pyrite, sphalerite, hematite and halite), with fungi (*Macrolepiota procera*, with its mycelium and spores),  plants (ostrich fern *Matteuccia struthiopteris,* with vitamin C, or ascorbic acid, crystal above; barley *Hordeum* sp.; cowslip primrose *Primula veris*; bush vetch *Vicia sepium*; lady’s mantle *Alchemilla vulgaris*; and wild strawberry *Fragaria vesca*), pollen (gazania pollen *Gazania* sp.*,* pumpkin pollen *Cucurbita* sp.; and birch pollen *Betula* sp.), insects (a bumblebee *Bombus* sp.; and the damselfly, *Calopteryx virgo*), cyanobacteria (*Prochlorococcus* and chain-forming cyanobacteria), diatoms (*Chaetoceros debilis; Surirella;* and *Licmophora flabellata*), plankton and other aquatic microorganisms (copepods of the order Calanoida; a dinoflagellate; ciliate protists including *Stentor coeruleus;* and green alga *Micrasterias*), fish (Atlantic salmon *Salmo salar*), seabirds (little auk, *Alle alle*) and mammals (reindeer *Rangifer tarandus;* humpback whale, *Megaptera novaeangliae*). The selected motifs reflect the paper’s movement from elemental composition and human–microbiome relations ("Elements in the human body" and "The human-microbiome-element symbiosis" sections) to microbial, atmospheric, hydrological, terrestrial, marine and animal mediation across the biosphere ("Elements in the human body mediated by organisms and ecosystems" section). The motifs are inspired by Scandinavian temperate ecosystems. The cyclic composition highlights multidirectional relations rather than a single, universal pathway or cycle. The human figure is based on dancer and choreographer Anna Asplind. A detailed guide to the motifs and source acknowledgements is provided in Supplementary Information, Fig. S1 (nos. 1–58).

Life has evolved by developing intricate ways of using a set of basic building blocks, the chemical elements available on planet Earth. These elements are continuously exchanged and transformed between living organisms and their surroundings, storing, transporting and making energy accessible for life. As life expanded and diversified, this exchange became integral to the dynamics and functioning of the Earth system, with organisms not only adapting to but also shaping and maintaining homeostasis of the fundamental chemical conditions of the atmosphere, the ocean, and Earth’s surface (e.g. Schlesinger et al. 2011; Lenton and Latour 2018). This interplay portrays the dynamic relations of complex adapting and evolving living systems, where system parts interact to form emerging patterns and dynamics that will feed back on those same parts in a continuous process (Levin 1998) (Fig. 1).

Here, we bring together knowledge from diverse areas into a synthesis of such co-evolved interdependencies of human bodies, elemental cycles, and the biosphere. In this way, we examine the material foundation of being intertwined with the planet and clarify the inseparability of human existence from the web of life and the chemical elements on planet Earth.

We focus on the role of basic living processes and relations—from microorganisms within the life processes of human beings to the significance of the web of life in mobilising, mediating, and transforming chemical elements vital for the functioning of the human body and being.

We first illustrate the significance of essential elements from the Earth system in relations and dynamics of human microbiomes and human bodily functions and place them in their evolutionary context. We then expand into the work of microorganisms, fungi, plants, and animals in ecosystems for the air we breathe, the water we drink, and the food we eat, shaping and mediating essential flows required for human health and wellbeing.

It is through these interrelations that humans have emerged as part of the evolution of life and continue to exist as ‘beings in the biosphere’ (Anderies and Folke 2024). Revealing such basic interdependencies within the biosphere has implications for how we understand the world, how knowledge is acquired, and how to act on it. It has implications for how to engage with human-environment relations and sustainability in the new trajectory of the Anthropocene (Steffen et al. 2007; Malhi 2017). It is in this context that we discuss the Anthropocene challenge of human alteration and reshuffling of essential life-element relations. The article highlights the foundational biogeochemical reality of being alive, of being able to sense, perceive, reflect, and value, of being inseparable, embodied, and enearthed (Schill et al. 2019) (Fig. 1).

## Elements in the human body

All living organisms are constituted of chemical elements as the basic building blocks of complex matter. Humans are no exception. Human life is made possible through the flows and cycles of these elements and their interplay with life in the biosphere, in unique and important ways. The human body can be described as an open, complex dynamic system that is embedded in the biosphere, and exists as part of biosphere-mediated elemental flows. Here, we focus on such flows within our bodies.

### Essential elements in the functioning of the human body

The human body contains at least 60 detectable chemical elements, with about 20 of these elements critical for basic metabolism in the human body (Chellan and Sadler 2015; Zoroddu et al. 2019). Six common elements dominate, together constituting 99% of human body mass—oxygen, hydrogen, nitrogen, carbon, calcium, phosphorus—forming the scaffolding of organic molecules and the structural material of bones, tissues, and cells. Another five elements—sulphur, potassium, sodium, chlorine (primarily as chloride), and magnesium—are critical for nerve conduction, muscle contraction, enzymatic function, fluid balance, and other core functions.

Water, made up of oxygen and hydrogen, is the human body’s most abundant chemical component, making up 50–60% of adult body mass (Lu et al. 2023) and some 74% of an infant’s body mass (Young et al. 2021). Every cell and organ in the human body needs water to function. Water is essential for the exchange of oxygen and carbon dioxide in the lungs, the filtering of waste and regulation of the electrolyte balance in the kidneys, and the transmission of signals between neurons that are necessary for generating thoughts and dreams in brains. Humans cannot survive without water for more than a few days (Lange and Schuster 1999).

In addition, trace elements such as cobalt, copper, iodine, iron, manganese, molybdenum, selenium, and zinc serve vital functions. They act as cofactors in enzymes, regulate hormonal processes, enable oxygen transport, and support immune defence and DNA transcription. Trace elements are simply indispensable for life. External sources are necessary as the body is unable to synthesise them (Mertz 1981; Moksnes et al. 2024).

Some elements, like calcium and phosphorus in bones, are stored over long periods, while others, such as magnesium and zinc, cycle through the body more rapidly—highlighting the different temporal rhythms of element use and turnover in the body (Table 1).

**Table 1:** Elements and key nutrients underpinning core human bodily functions. Rows are grouped broadly into movement and signalling, structure and regulation, growth and reproduction, and cellular maintenance. Parentheses indicate either key associated element(s) (e.g. Co in vitamin B12) or, for organic nutrients, the major elements in their chemical composition (e.g. CHO; CHON)

Bodily function	Associated nutrients (and key elements)	Primary roles
Heartbeat (Heart)	Potassium (K), calcium (Ca), sodium (Na), magnesium (Mg)	Regulate heart rhythm, muscle contraction
Brain function and development	Magnesium (Mg), zinc (Zn), omega-3 fatty acids (CHO)	Cognition, synaptic plasticity, and neurodevelopment
Signalling (Nervous system)	Potassium (K), sodium (Na), chloride (Cl), calcium (Ca)	Transmit nerve impulses and maintain electrochemical gradients
Movement (Muscles)	Magnesium (Mg), amino acids (CHON), calcium (Ca), potassium (K)	Muscle contraction, repair, metabolism
Structure and movement (Bones)	Calcium (Ca), magnesium (Mg), phosphorus (P), vitamin D (CHO), vitamin K (CHO)	Build and maintain bone matrix and skeletal function
Circulatory system (Blood)	Iron (Fe), water (H_2_O), vitamin B12 (Co), folate (CHON), copper (Cu)	Oxygen transport, red blood cell formation, blood volume and circulation
Hormone regulation (Thyroid)	Iodine (I), selenium (Se)	Regulate metabolism, growth, and hormone production
Temperature regulation (Water balance)	Sodium (Na), water (H_2_O), chloride (Cl), potassium (K)	Maintain fluid balance and thermoregulation
Immune defence	Zinc (Zn), Vitamin C (CHO), selenium (Se), iron (Fe)	Support immune responses and host defence
Blood clotting	Vitamin K (CHO), calcium (Ca)	Enable blood coagulation
Labour and reproduction	Iodine (I), iron (Fe), zinc (Zn), selenium (Se), calcium (Ca)	Fertility, endocrine regulation, and smooth muscle contraction
Pregnancy and foetal development	Folate (CHON), iron (Fe), iodine (I), vitamin A (CHO)	Support neural tube development and foetal growth
DNA synthesis and repair	Phosphorus (P), folate (CHON), vitamin B12 (Co)	Nucleotide synthesis, methylation, cell division
Energy production	Magnesium (Mg), B vitamins, iron (Fe)	Facilitate ATP production in mitochondria
Cell membrane and structure	Phospholipids (CHOP), choline (CHON), omega-6 fatty acids (CHO), omega-3 fatty acids (CHO)	Maintain cellular structure and integrity
Antioxidant defence	Vitamin C (CHO), vitamin E (CHO), selenium (Se), zinc (Zn)	Protect cells from oxidative damage

The elements compose all of life’s foundational molecular groups—proteins, carbohydrates, lipids, and nucleic acids—and contribute to physiological regulation across every major bodily system. Amino acids, fatty acids, and vitamins are composed of these elemental building blocks.

The chemical elements each play multiple roles in the body, ranging from the development, operation, and dynamics of our organs to the functioning of microbes in the human body, to the chemical communication central in the cellular system that allows the translation of environmental conditions into appropriate reactions and adaptations (e.g. Gschwind et al. 2001; Silpe and Balskus 2021). Their transformation and transport also help store accessible forms of energy. Healthy heart rhythm, neural conductivity, immune function, and metabolic regulation all rely on the availability and balance of specific elemental combinations.

Although our bodies may appear to be persistent structures, in fact the body’s elemental composition is in dynamic flux, regulated through processes of ingestion, transformation, absorption, storage, and excretion. The functioning of the human body is reliant on the continual intake, circulation, and regulated use of the biologically essential elements and thereby dependent on biosphere-mediated elemental cycles.

## The human-microbiome-element symbiosis

Microbes (bacteria, archaea, fungi, protists, viruses) are an essential part of us, entangled with the rest of our cells as part of a highly diverse cellular community. Over half the cells in a healthy human body belong to the multitude of species that compose our microbiome. Hence, human bodies are microbial habitats (Sender et al. 2016; Gilbert et al. 2018).

Human microbiomes perform functions that are integral to whole-organism health, including governing the uptake of essential elements (Bielik and Kolisek 2021), influencing not only physical health but also emotion, thought, and behaviour (Bosch et al. 2024).

This integration of host and microbiome is referred to as the holobiont (Margulis and Fester 1991; Robinson et al. 2026) and typically includes a eukaryote host and all of the viruses, bacteria, fungi, etc. that live on or inside it—many of them symbiotic. Together they function as a metaorganism, operating complex metabolic processes and mediating the cycling of an associated array of chemical elements (Bosch and McFall-Ngai 2011).

Human microbiomes are intimately involved in host biochemistry. The gut microbiome, for example, supports digestive processes such as the breakdown of proteins into amino acids, and the conversion of complex compounds into bioavailable forms of essential elements, such as phosphorus and sulphur (Beane et al. 2021; Barone et al. 2022). At the same time, microbial communities compete with host cells for metals and other elements needed for enzymes and electron transfer (Bielik and Kolisek 2021). Human bodies as hosts conduct tight elemental regulation, especially of iron and other micronutrients that microbes require, so that immunity and nutrition rely in part on the regulation and exchange of limiting elements between host and microbial cells (Egan et al. 2024).

Human-microbiome life processes and relations reflect our evolutionary origins of being alive today. A few examples follow.

### The gut microbiome

Life on Earth originated some 3.7 billion years ago in an environment under low oxygen conditions. The earliest life forms were slow-growing obligate anaerobes that used nitrate, fumarate, sulphate, or sulphur as electron acceptors instead of molecular oxygen to release energy (Canfield et al. 2006). It is fascinating that metabolically similar anaerobic bacteria exist within our bodies today and constitute by far the most common class of microorganisms inhabiting the near-oxygen-free environment of the large intestine. There they fulfil vital functions, such as digestion of dietary compounds, synthesis of key nutrients, and mucosal immune regulation (Skrypnik and Suliburska 2018; Barone et al. 2022). They also mediate the flow of elements to and from the human host––influencing the absorption of key minerals, production of bioactive metabolites, and synthesis of essential vitamins––with substantial consequences for whole-organism health (Maier et al. 2015; Rowland et al. 2018; Mann et al. 2024). This continuity reflects our deep-time evolutionary entanglement with the origin of life and chemical elements of planet Earth.

Some 3000 species of bacteria have been reported to live in the human gut (Wan et al. 2023). These microbial communities shape our physiology through numerous functional pathways. They act as gatekeepers for essential elemental flow by influencing intestinal barrier integrity and producing metabolites that regulate mineral absorption pathways, thereby shaping which nutrients enter systemic circulation in the body.

The microbial production of short-chain fatty acids (SCFAs) in the gastrointestinal tract illustrates the interconnections of chemical elements, body functions and the gut microbiome (e.g. Yilmaz and Li 2018; Beane et al. 2021). Essential inorganic minerals, such as calcium, magnesium, iron, and phosphorus, must be acquired from environmental and dietary sources (Skrypnik and Suliburska 2018). This is where the metabolic activity of the gut microbiome plays a key role in breaking down dietary fibres (e.g. nuts, seeds, fruits, and unprocessed whole grains)—otherwise indigestible by the host—into SCFAs such as butyrate which is a primary energy source for our intestinal epithelium and support gut health (e.g. Kiela and Ghishan 2016; Rowland et al. 2018; Bielik and Kolisek 2021; De Vos et al. 2024).

Beyond these local effects, SCFAs such as butyrate, acetate, and propionate function as key signalling molecules in the microbial gut-brain axis (Dalile et al. 2019; Sharma et al. 2021; O'Riordan et al. 2022). These highly bioactive microbial metabolites can influence a range of psychoneuroimmunological and endocrinological outcomes through effects on epithelial barrier integrity, immune responses, hypothalamic–pituitary–adrenal axis activity, and the synthesis of neuroactive compounds such as serotonin (e.g. Wu et al. 2020; Cheng et al. 2024).

Group B vitamin metabolism within the human gastrointestinal tract provides another well-characterised example of essential nutrient exchange between resident microbial communities and the human host. These vitamins are vital for multiple core physiological processes, including DNA and neurotransmitter synthesis, hormone regulation, energy metabolism, and immune function (Hossain et al. 2022). However, human cells alone do not synthesise sufficient quantities of these compounds, necessitating additional sources through microbial biosynthesis in the gut (Magnúsdóttir et al. 2015), including thiamine (B1), riboflavin (B2), niacin (B3), pantothenic acid (B5), biotin (B7), folate (B9), and cobalamin (B12) (Biesalski 2016). For example, up to 37% of the daily folate requirement (B9)—an essential nutrient in nucleotide synthesis and epigenetic modification (Ebara 2017)—in healthy adults may be supplied by gut bacteria, particularly by members of the Bacteroidetes, Fusobacteria, and Proteobacteria phyla (e.g. Magnúsdóttir et al. 2015). Some gut bacteria also encode the biosynthetic pathways for menaquinone (K2), a microbially derived form of vitamin K that serves as an essential cofactor in blood clotting (Tarracchini et al. 2025).

The gut microbiome composition helps prevent colonisation by harmful pathogens that rely on oxygen and contributes to immune system regulation. It is hypothesised to influence health outcomes, mood, stress management, and even dietary choices (Griffiths et al. 2026). It has been estimated that the human microbiome is involved in a dialogue between 71% of faecal and 15% of blood metabolites (Visconti et al. 2019).

### The skin as a biological filter

The body’s skin is an evolved interface where elemental chemistry and microbial communities jointly regulate exchange between the body and the external environment. The polymer melanin, composed of carbon, hydrogen, nitrogen, oxygen and sulphur, helps absorb harmful UV radiation (Cordero and Casadevall 2020). The keratinised barrier of the skin is built from sulphur-rich structural proteins and lipids, while sweat and sebaceous secretions regulate salt, water, and pH at the surface. Small but consequential trace elements (notably zinc and copper) support barrier integrity, collagen formation, and wound repair, and metal availability is tightly managed because skin-associated microbes also require these elements to grow. The skin microbiome, in turn, helps set local chemistry (e.g. acidity) and competes with opportunists, so elemental conditions on the surface become part of how the body maintains a stable interface with its surroundings (Yong 2016). Our skin is home to millions of bacteria, archaea, fungi and viruses that compose the skin microbiota. These microorganisms play essential roles, for example, in defence against invading pathogens and the education of the immune system (Byrd et al. 2018).

### A co-evolutionary relation

The transfer of essential vitamins from microorganisms to the human host is not unidirectional but reflects a broader network of exchange between them (Sharma et al. 2019). Host- and diet-derived elements provide substrates that sustain microbial growth and metabolism, thereby shaping the composition and functional capacity of resident microbial communities. In turn, the activity of these communities influences the chemical forms and availability of vitamins and minerals that are required to maintain host physiological function (Barone et al. 2022). Such shared dependencies give rise to an interconnected metabolic network in which vitamin availability, microbial community structure, and host physiology and diet are tightly linked. This reflects co-evolution.

While the gastrointestinal tract provides the most extensively studied example of a co-evolved relationship, there is evidence that microbial communities at other body sites are also engaged in similar processes. For example, the oral microbiome includes commensal members that play key roles in maintaining oral health (Pascual et al. 2023). One critical process involves the regulation of pH within dental plaque and saliva, which directly governs the solubility of calcium-phosphate minerals in tooth enamel (Strużycka 2014). Some oral commensal bacteria produce alkaline metabolites that buffer against acidification, thereby limiting enamel demineralisation by stabilising calcium and phosphorus—vital structural components of teeth—within the mineral matrix and protecting against tooth decay. These alkali-generating pathways, including arginine and urea metabolism, generate ammonia that raises local pH and provides bacteria with energy that is essential for their survival (Nascimento et al. 2013).

Microbes influence the bioavailability and chemical form of nutrients and trace elements by breaking down complex food matrices, transforming nitrogen- and sulphur-containing compounds, liberating phosphate from organic matter, and competing for metals needed for enzymes and electron transfer. Human bodies as hosts respond with tight elemental regulation—especially of iron and other micronutrients that microbes require—so that immunity and nutrition become partly a matter of controlling access to limiting elements. Such dynamics involving oxygen, iron, and bacteria in the human body are presented in the Supplementary information.

Humans use oxygen to break down glucose to generate energy. This process is carried out by mitochondria, membrane-bound cell organelles that convert nutrients from food into adenosine triphosphate (ATP) and power the cell's biochemical reactions. Mitochondria evolved through a process called endosymbiosis, where a free-living aerobic bacterium was engulfed by another host cell unable to utilise oxygen (Fig. S1, nos. 10–12). The bacterial symbiont thereby provided the host with a more efficient way to produce energy through aerobic respiration (e.g. Geiger et al. 2023). Our mitochondria can still divide by fission. They contain their own circular DNA, as bacteria do, and have their own ribosomes that are more like those of bacteria than the ribosomes of our host cells (e.g. Boguszewska et al. 2020).

These few examples illustrate the intricate dynamics of human body functioning and the human microbiome, and their co-evolutionary origins with microbial life in the biosphere. The role of microbes in mediating essential element cycles in humans reflects an extension of the inseparable relations of human life with the wider ecological and biogeochemical systems of planet Earth.

## Elements in the human body mediated by organisms and ecosystems

All life is sustained by energy and elemental flows. In this section, we explore our intertwined relations with life across the planet—from microbes to mammals—in mediating the cycles of chemical elements that are critical for the dynamic life processes within our bodies. Mediation by life encompasses the steps through which elements are released from reservoirs, made bioavailable, transported, incorporated into living structure, and eventually returned to the environment.

The dynamic and complex life processes of our bodies, just like those of other living animals, acquire essential chemical elements through air, water, and food. Bodies make use of them in life-sustaining processes and relations that incorporate them in diverse forms into tissues and cells. Eventually, they leave the body through excretion, perspiration, respiration, or decay, making elements available for recycling pathways through which microorganisms, fungi, plants, and animals again transform and concentrate elements into bioavailable forms. Such life dynamics tie human metabolism to the broader cycles of the elements within the biosphere (Fig. 1).

Life’s relation with carbon is profound. Carbon serves as a backbone, the building block of life on Earth. It is a core component of all complex biological molecules of life, like proteins, lipids, nucleic acids, and carbohydrates and is fundamental to human physiology like respiration and metabolism, and to diet. Carbon cycles through organisms, the atmosphere, and the ocean, and the life-carbon interplay forms soil, regulates Earth's climate, and sustains foundational conditions of a liveable biosphere for humanity (Steffen et al. 2004; Rockström et al. 2021).

In living organisms, energy is stored in chemical bonds, particularly in carbon-based molecules, whose bonds are sufficiently stable to persist and form the molecular basis of life, yet weak enough that they can be broken and reformed in controlled ways. In this sense, photosynthesis and respiration are inseparable parts of a single cycle: photosynthesis stores solar energy in organic matter, while respiration releases that energy to sustain life processes across organisms, humans included.

This energetic coupling is the result of deep evolutionary history. Ancient respiratory bacteria became incorporated into cells as mitochondria, and photosynthetic cyanobacteria became incorporated as chloroplasts in algae and plants. Together, such evolutionary innovations link photosynthesis and respiration to a planetary-scale metabolic cycle that regulates atmospheric composition, stabilises the climate system, and underpins the emergence and persistence of complex life on Earth (e.g. Lenton et al. 2016).

Plants, algae, and cyanobacteria are the principal photosynthetic producers on planet Earth (Fig. S1, e.g. nos. 2, 5–8, 14, 19–23, and 29–30). Powered by solar energy, they, through the process of photosynthesis, convert water and carbon dioxide into oxygen and carbohydrates, thereby providing oxygen for respiration and organic matter that forms the basis of food webs on which other living organisms, humans included, depend. Animals consuming plants (human and non-human, e.g. Fig. S1, nos. 1 and 15) acquire and bioaccumulate essential elements, which can then move through food webs and, in some cases, become concentrated at higher trophic levels. Dead plants and animals are decomposed by detritivores and microbes into soil and sediments (Fig. S1, nos. 31 and 33–34); basic elements are released and become available again for plants and algae and the cycles continue. Life is renewed, develops, and evolution continues. Water plays a central role throughout these life processes; hence it can be considered the bloodstream of the biosphere (Falkenmark and Biswas 1995). Humans and our bodies are tightly coupled with these dynamics of life.

**Fig. 2:**
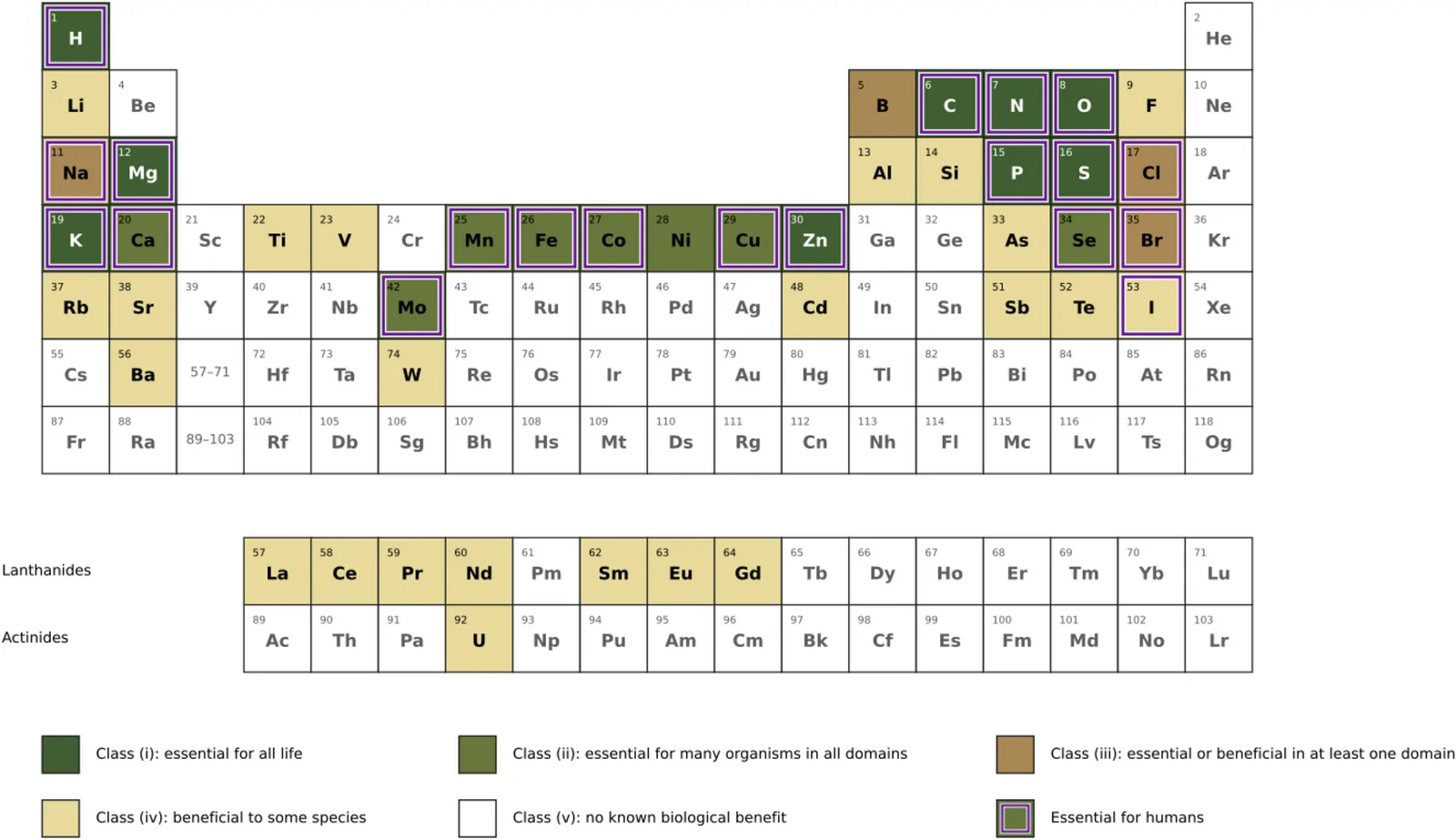
**Life and the periodic table**. Chemical elements are shaded according to their documented biological roles. Class (i) elements, shown with the darkest fill, are essential for all life and include the bulk macronutrients CHNOPS, two additional elements commonly present as ions (K, Mg), and one transition metal (Zn). Class (ii) elements are essential for many organisms across all three domains of life. Class (iii) elements are essential or beneficial for many organisms in at least one domain. Class (iv) elements are beneficial to at least some species. Class (v) elements, shown with a white background and grey symbols, have no known biological benefit. Elements known to be essential for humans are indicated by an inset double border, with a white halo and purple inner line. Adapted from Zoroddu et al. (2019) and Remick and Helmann (2023)

Life’s evolved relationships with the biogeochemical cycles of all six key elements carbon, hydrogen, nitrogen, oxygen, phosphorus, and sulphur (abbreviated as CHNOPS) build and sustain life and shape the biosphere and Earth System dynamics (Steffen et al. 2004). These macronutrients (CHNOPS) together with two elements commonly present as ions (potassium, magnesium) and one transition metal (zinc) are essential to all life on Earth from bacteria to mammals, humans included. An additional eight elements are critical for many organisms across all domains of life (calcium, cobalt, copper, iron, manganese, molybdenum, nickel, selenium) (Remick and Helmann 2023), reflecting the pervasive, interwoven, and inseparable relationship of life with the chemical elements on Earth (Kaspari 2021) (Fig. 2).

Microbial metabolism altered the redox state of the atmosphere and oceans, plants co-evolved with fungal partners to mobilise minerals and restructure soils, and animals evolved mobility that enabled the long-distance redistribution of nutrients. In each case, the co-evolution of life and the geochemical environment gave rise to new ways of mobilising, activating, transporting, assembling, and recycling the elements.

Micronutrients like selenium and iodine may trace paths from distant marine sediments and mineral reservoirs to crops and dietary supplements through to human tissues. Other elements, like nitrogen and phosphorus, cycle through agricultural systems that are mediated by microbial metabolism. Water serves as a carrier of dissolved ions such as magnesium and fluoride and is also consumed in enzymatic hydrolysis during digestion. Oxygen is absorbed directly through respiration.

### Microorganisms in the human-elements interface

The biosphere’s elemental cycles pass through human beings, and microorganisms act as essential biological agents in this process, integrating Earth’s geochemistry into living organisms (Dong et al. 2022). Microorganisms have been evolving for at least 3.5 billion years. Microbial life represents the majority of Earth’s biodiversity and microbiomes operate in symbioses that are vital to macro-scale processes in plants, animals and entire ecosystems (Averill et al. 2022). In fact, microbiomes serve as preconditions for the existence and survival of their hosts whether plants, animals or humans.

The microbiome-host symbiosis is increasingly described as ‘metaorganismic’, referring to the totality of any multicellular organism derived from millennia of co-evolution with microbiota. A metaorganism is the host and its synergistic interdependence with bacteria, archaea, fungi, and numerous other microbial and eukaryotic species including algal symbionts (Bosch and McFall-Ngai 2011).

Through their metabolic activities, microbes change elemental reservoirs into biologically accessible forms, continuously reshaping biogeochemical landscapes and seascapes, and enabling the emergence and persistence of complex life (e.g. Amachi 2008; Moran and Durham 2019; Duhamel 2025). For example, heterotrophic bacteria are the only organisms in marine ecosystems that efficiently assimilate and transform dissolved organic matter, a process of great significance given that photosynthetic cyanobacteria and single-celled eukaryotic phytoplankton (Fig. S1, e.g. nos. 14, 18–22 and 29–30) carry out roughly half of the primary production on Earth with some 50% of the organic carbon produced channelled through heterotrophic bacteria (Bunse and Pinhassi 2017).

Many of the elements necessary for human survival are abundant in the minerals and rocks (e.g. Fig. S1, nos. 41–54), soil, air, water and organic debris that surround us. Yet they are largely unusable by our bodies in these forms. Atmospheric nitrogen exists predominantly as inert N_2_, with a triple bond that cannot be broken by human metabolism. Rocks contain sequestered phosphorus and sulphur that we cannot access. Microorganisms bridge these and other gaps, converting elemental pools into forms that can enter the food web, and ultimately our bodies. For instance, microbes convert atmospheric nitrogen into bioavailable ammonia (NH_3_) through biological nitrogen fixation, followed by microbial transformation of ammonia into nitrite and nitrate that can be assimilated by plants, and subsequently human beings through diet. Microbes also regulate iron availability through redox transformations, decompose biomass into organic acids, and play a central role in the sulphur cycle through the oxidation of sulphur compounds (Fig. S1, no. 49) into sulphate (Zaman et al. 2025).

Clearly, microbiome-host symbioses do not only play essential roles in the life-element relationships within us humans but also across the whole tree of life of eukaryotes.

### Phytoplankton, plants and the air we breathe

In every breath of air that you take, up to twenty every minute, vital oxygen (O_2_, constituting ~ 21% of air) fills the lungs. The oxygen passes from the lungs into the blood and is circulated through the workings of the heart to all tissues and organs of the body, where our cells use it to transform nutrients into usable energy.

About half of the oxygen we breathe comes from ocean ecosystems, produced as a byproduct by algae capturing sunlight. This happens in the photic zone, extending from the ocean surface down to some 200 m, in algae ranging from large kelp forests of coastal ecosystems to microscopic free-floating phytoplankton. But the dominant part of ocean oxygen is produced by phytoplankton, such as diatoms and cyanobacteria. Diatoms (Fig. S1, e.g. nos. 14, 19–20 and 39), tiny, single-celled phytoplankton, are essential mediators of the oxygen cycle (Alverson et al. 2025). Just one species, the cyanobacterium *Prochlorococcus marinus (*Fig. S1, no. 21)—the smallest and most abundant photosynthesising picoplankton in the open ocean—is responsible for as much as 5% of global photosynthesis (Chisholm et al. 1988; Biller et al. 2015).

The other half of oxygen production occurs in terrestrial ecosystems. Here, tropical forests contribute some 34% of the terrestrial oxygen production. The Amazon forest alone generates about 16% of terrestrial oxygen (corresponding to about 9% of global oxygen production by ecosystems) (Y. Malhi, University of Oxford, pers. comm.). Tropical savannahs and grasslands generate 26%, temperate and boreal forests some 15% and croplands about 12% of the terrestrial oxygen; the remainder is generated by temperate grass and shrublands, deserts, and tundra (based on Beer et al. 2010).

However, the bulk of the oxygen in the atmosphere is not set by the activities or existence of current biomes. In fact, the biomes of the ocean and on land use up, mainly through respiration, about the same amount of oxygen as they produce (i.e. net zero) (Field et al. 1998). The oxygen levels in the atmosphere are rather set on million-year timescales by the subtle balance of geological, chemical, and biological processes, where oxygen has accumulated in the atmosphere due to the burial of organic matter and pyrite in sediments (Fig. S1, no. 42), at a rate four times higher in the ocean than on land. Consequently, over geological time some 86% of the oxygen content of the atmosphere has come from the ocean, implying that the oxygen in more than six out of seven breaths taken by humans is ocean-derived entangling us with life over vast timescales (Grégoire et al. 2023). In broad terms, the ocean has contributed nearly all the oxygen in the atmosphere from the time of cyanobacterial evolution, around 2.4 billion years ago.

Hence, through oxygen, humans are not only intertwined with the current ecosystems of the biosphere but also with the work of life across deep time.

### Water we drink and the bloodstream of the biosphere

Water (H_2_O) is the fluid of all living organisms and essential for the human body. Since water cannot be stored in the human body, a continuous turnover of 1–6 L daily is needed for proper body functioning (Yamada et al. 2022).

Water is crucial in transporting, assembling, mobilising, activating, and recycling life-essential elements (Falkenmark et al. 2019). The patterns and dynamics of freshwater’s continuous circulation are shaped not only by the Earth's atmosphere, plate tectonics, topography, and the sun’s radiation, but also by living ecosystems that store soil moisture, uphold evaporation, and sustain downwind rainfall.

Through the water cycle, essential basic elements like calcium and magnesium (up to 20% of the total daily intake), potassium, and trace elements enter the human body (WHO 2005). In the human body, water continues to act as a solvent, reactant, and carrier, while the large heat capacity of water allows it to act as a thermoregulator. In addition, water forms a lubricating fluid for the joints and digestive and respiratory tracts and helps absorb shocks from walking and running (Jéquier and Constant 2010) and flush many elements out of our bodies. Through the water that flows into the human body and leaves the body, we become an inseparable part of the dynamics of the Earth’s hydrological and biogeochemical cycles.

Around 50–80% of daily human intake of water comes from what we drink (e.g. Tani et al. 2015). Globally, most of the drinking water is today sourced from groundwater or surface water in reservoirs, lakes, and rivers, while locally important sources also include rainwater harvesting, and springs, as well as desalinated seawater. Freshwater is made available to humans through processes driven by energy from the sun, the gravity of the Earth, and the activities of living ecosystems.

The ocean evaporates some 85% of global water vapour, and some 20% of global water vapour falls again as rain over land (van der Ent et al. 2010; Vargas Godoy et al. 2021). On land, rainfall sustains soil moisture, replenishes groundwater, runs off into streams and rivers and eventually makes its way back to the ocean. Terrestrial ecosystems intercept precipitation in their canopies, litter, and stems, while plants transpire water vapour into the atmosphere, a process closely coupled to photosynthesis. Around 40–50% of the precipitation over land—and substantially more in some seasons and regions—is recycled by evapotranspiration from soil and plants to the atmosphere (van der Ent et al. 2010; Tuinenburg et al. 2020). A barren world would generate less than one-third the evapotranspiration and half the land precipitation of a fully vegetated planet (Kleidon et al. 2000), making plants—and the evolutionary processes that shape them—a critical link between soil moisture and the atmosphere, and an important regulator of runoff (Savenije 2024).

Freshwater on land dissolves elements from rocks and absorbs minerals from soil, enriching the water we drink from rivers, lakes, reservoirs, and groundwater with essential elements like calcium, magnesium, and iron. Along its way, both terrestrial and aquatic ecosystems, such as forests, grasslands, and wetlands, actively modify the velocity, pH, and mineral content of the water.

The other 20–50% of human water intake comes from the food we consume, making food an important source of water (Tani et al. 2015). In general, the water content of fish, seafood, and vegetables is typically about 80–90% water (e.g. Van Ruth et al. 2014; Guelinckx et al. 2016).

Beyond the water human obtain through food, the production of food consumes the largest volumes of freshwater on which we depend. An adequate diet per person and day requires 3000–4000 litres of green water (evapotranspiration) (Rockström et al. 2009) compared to the 1–5 litres per person of daily water intake from food and drinks depending on environmental and lifestyle factors (Yamada et al. 2022). Further, food production depends on complex hydrological processes linking ecosystems and the water cycle. For example, terrestrial moisture teleconnections are fundamental for rainfed agriculture, where croplands in as many as 155 countries receive up to 40% of their annual precipitation from moisture derived from forests located in other countries and sometimes far away (Pranindita et al. 2025).

Moreover, virtually all minerals that enter the human body have been mediated by water through transport, assembly, mobilisation, activation, and recycling directly via the water cycle itself or indirectly via water’s role in enabling life processes and in regulating climate and weather. Water absorbed by plant roots transports essential elements from soil and sediments to the rest of the plant. Many freshwater plants acquire essential dissolved elements from the water column through the leaves. Freshwater fish absorb ions from the water through their gills and excrete excess water through dilute urine, while marine fish drink seawater, and excrete excess salts through their gills and concentrated urine.

The world we experience today—including the evolution of humanity itself—is very much the result of a co-evolutionary dance in which water shaped life and life shaped water over the 3.5 billion years of biosphere history. Thus, water is a non-negotiable prerequisite for our existence, serving life-ensuring functions within our bodies and entangling us with the essential elements and life processes in diverse ecosystems across the continents, the ocean, and the atmosphere. It demonstrates how deeply intertwined humans are with water, the “flowing bloodstream” of our unique biosphere (Falkenmark et al. 2019).

### Soil, plants, and elements in the food we eat

Life in soil is foundational for elemental cycling in terrestrial food production. Soil organisms, like bacteria, fungi, archaea, protists, plants, and animals (Fig. S1, e.g. nos. 23, 31, 33–34 and 55–58), determine the turnover of the organic matter pool in soils and perform significant work in breaking down organic matter (decomposition) and cycling and converting essential elements (mineralisation) into forms that become available for plant growth in all ecosystems.

Soil is a vast habitat for different organisms and soil biodiversity represents almost 60% of the species on Earth, from microbes to mammals (Anthony et al. 2023). A gram of soil may contain up to 10 billion microorganisms of possibly thousands of different species (Torsvik and Øvreås 2002). Differences in the structure of soil communities can drive large variation in elemental cycling (Crowther et al. 2019).

Soil stores a major part of the organic matter in the terrestrial biosphere, with more carbon than vegetation and the atmosphere combined (Crowther et al. 2019). Soil animals (detritivores), like worms and mites, decompose dead plant materials and animal waste. Earthworms for example create a more favourable environment for plant roots, making it possible for plant roots to grow deeper and access more water and nutrients (van Groenigen et al. 2014). They also play an important role in soil nitrogen cycling (Lang et al. 2023).

Most elements that plants need, such as nitrogen, phosphorus, potassium, and sulphur, are bound in organic molecules or minerals that are seldom directly available for plants. Plant growth is therefore dependent on soil microbes such as bacteria and fungi, which possess the metabolic machinery to depolymerise and make available organic and insoluble mineral forms of essential elements. Such microbial transformations are essential for all plants (Jacoby et al. 2017; Oyarte Galvez et al. 2025).

Plants in turn shape microbiome structures, most likely via root exudates, and bacteria have developed adaptations to prosper in the rhizospheric niche (the soil associated with plant roots), a hotspot of ecological richness, with plant roots hosting an enormous array of microbial taxa (Jacoby et al. 2017; see Fig. S1, nos. 23 and 34). In addition, mycorrhizal fungi form symbiotic associations, based on the exchange of resources, with the roots of many plants, extending the foraging reach of plants through hyphal networks that access pore spaces and mineral surfaces unavailable to roots, shaping the elemental composition of plant tissues. The plant receives soil nutrients from the fungus, and the plant provides sugar as a source of carbon to the fungus. In a mutualistic fashion, plants detect, favour, and reward the best fungal associates with more carbohydrates and their fungal partners enforce cooperation by strengthening nutrient transfer only to those roots providing more carbohydrates (Kiers et al. 2011). Some mycorrhizal fungi rely on inorganic forms of nitrogen that have been mineralised by free-living decomposers, whereas others can degrade and acquire organic forms of nitrogen directly, trading nutrients for plant carbon and shaping the elemental composition of plant tissues (e.g. Lambers et al. 2009; Mishra et al. 2023).

Nematodes (Fig. S1, no. 33) form an important part of soil biodiversity as abundant and functionally diverse animals affecting plant performance (Wilschut and Geisen 2021). The feeding by nematodes on bacteria and fungi keeps these communities in an active growth phase. In addition, the movement of nematodes spreads nutrient-rich bacterial and fungal propagules, thereby stimulating bacteria- and fungi-mediated mineralisation in soil (Schratzberger et al. 2019; van den Hoogen et al. 2020; Li et al. 2024).

Soil microbes importantly facilitate the transport of essential trace minerals, such as zinc, iron, copper, and selenium, into plants where they contribute to enzyme function and participate in photosynthesis and respiration. Trace minerals from the soil (Fig. S1, e.g. nos. 41–43, 45 and 51) enhance the nutritional quality of, e.g. crops (Mishra et al. 2023), vegetables, and fruits (Ahmed et al. 2024) and become further concentrated in organisms that consume them.

The micronutrient density of crops, wild plants, and tree foods reflects rhizosphere interactions and the integrity of soil food webs that release, transform, and transport elements through microbial growth and grazing. Fungi offer a particularly direct illustration: edible mushrooms (Fig. S1, nos. 31–32) are the fruiting bodies of extensive mycelial networks, and many species draw trace elements, such as selenium, zinc, iron, and copper from their substrates (e.g. Denton-Thompson and Sayer 2022).

The soil fungal network can increase plant biomass by 20–50%, water-use efficiency by up to 60%, and reduce heavy metal uptake in plant tissue by 30–70%. Since our DNA relies on phosphorus, and most plants require mycorrhizal fungi to absorb it from the soil, it can be inferred that a large portion of the phosphorus in our DNA has passed through a mycorrhizal fungal network (Kiers 2026).

Furthermore, microbes and fungi can accelerate mineral weathering by exuding organic acids and metal-binding ligands that dissolve otherwise insoluble phases and keep micronutrients in solution. They also mediate redox transformations that change solubility and mobility (e.g. iron and manganese cycling), shifting which elemental forms are accessible to roots (e.g. Ribeiro et al. 2020).

Clearly, soil organisms perform often unrecognised work that makes it possible for us humans to receive, through the food we consume, the vital elements and trace minerals that our body requires. In this sense, we are deeply entangled with life in the soil for health and wellbeing.

### Marine ecosystems and elements in the food we eat

Phytoplankton and other primary producers convert dissolved inorganic forms into biomass, shaping the elemental composition of marine life (e.g. Alverson et al. 2025, e.g. Fig. S1, nos. 13 and 40) and seafood for humans (e.g. Golden et al. 2021; Crona et al. 2023). As such, they are a major entry point for elements into food webs.

Upwelling, as in the Benguela, Humboldt, and California current systems, brings essential macronutrients (e.g. fixed N, P, Si) and trace elements (e.g. Fe, Co, Mn, Cd, Ni, Cu) from deep waters or shelf sediments into the sunlit surface ocean fuelling high primary productivity of marine ecosystems and supporting marine life and fisheries (e.g. Cury et al. 2000; Liu et al. 2022). Seasonal mixing, atmospheric deposition, and episodic inputs can shift trace element availability and, in turn, alter biological uptake patterns.

Furthermore, the seafloor is simultaneously a habitat, carbon store, and biogeochemical processor, with burrowing animals and microbes involved in regulating how nitrogen, phosphorus, and carbon move between sediments and the overlying water (e.g. Snelgrove et al. 2018).

In many regions, biological production is constrained not only by macronutrients (such as nitrogen and phosphorus) but also by trace elements that act as enzymatic cofactors (for example, iron, zinc, cobalt, and manganese). For example, the productivity of much of the surface low-latitude ocean tends to be nitrogen-limited, but in upwelling regions iron is often a limiting micronutrient (Moore et al. 2013). This matters for human bodies because seafood is not only protein: it represents a concentrated route of bioaccumulation of essential elements in food webs through which marine biogeochemistry becomes human micronutrition.

Iodine, an essential constituent of thyroid hormones, is a special marine case. In the iodine-rich marine environment, iodine becomes concentrated in the biota, with particularly elevated levels in some edible seaweed. However, phytoplankton, especially diatoms, are strong concentrators of iodine and iodine accumulation by phytoplankton may be considerably greater than that of macroalgae (Fuge and Johnson 2015). Importantly, iodine is predominantly found in the oceans and therefore a critical dietary source of iodine for humans is through the consumption of seafood, such as fish (e.g. haddock, cod), shellfish and seaweed (kelp, nori, and kombu in particular).

Hicks et al. (2019) estimated how environmental and ecological traits predict nutrient content of marine finfish species, by compiling nutrient composition data across hundreds of marine fish species (including calcium, iron, zinc, selenium, vitamin A, and omega-3). They demonstrate that nutrient concentrations are linked to ecological traits and thermal regimes, and that there is no simple relationship to total fishery yield. These findings highlight that, for human consumers, the nutrient quality of a fishery is determined by its species composition and the work of marine food webs that produce them.

The same ocean processes that shape nutrient content also shape where the elements accumulate and where they are returned—often through the movement and metabolism of animals, which becomes explicit in the next section.

Ocean currents and circulation transport essential elements, which are captured by microorganisms, like phytoplankton and bacteria, thereby entering marine foods. Marine food webs bioaccumulate essential elements and make them available to humans through the seafood that we harvest for direct consumption (Hicks et al. 2019) or for production of fishmeal serving aquaculture like shrimp and salmon farming (Naylor et al. 2021; Elsler et al. 2026).

The vital elements and trace minerals that build our bodies through the seafood we eat make us humans interwoven with ocean processes and marine food webs in diverse ways.

### Elemental transport mediated by insects, birds, mammals, and migratory animals

For many human-essential elements, biosphere-scale fluxes are often described through plant, microbial, fungal, and abiotic processes. At the same time, animal movement and metabolism can be disproportionately important drivers of redistribution—moving nutrients across vertical gradients, ecosystem boundaries, and landscapes, thereby shifting local cycling rates and nutrient ratios (Schmitz et al. 2018). They act as “mobile links” and are important components in the dynamics of ecosystem development and ecosystem resilience (Nyström and Folke 2001; Lundberg and Moberg 2003; Bauer and Hoye 2014).

The significant role of pollinators moving across landscapes in enhancing crop production has long been appreciated and demonstrated (e.g. Buchmann and Nabhan 1997; Adamidis et al. 2019, e.g. Fig. S1, nos. 4 and 25–26). Pollinator-dependent crop species encompass fruit, vegetable, seed, nut, and oil crops, which supply significant proportions of micronutrients, vitamins, and minerals in the human diet (Potts et al. 2016). Pollination can reduce micronutrient deficiency in vulnerable communities as shown among smallholder communities in Nepal. Here, insect pollinators like native honeybees, bumblebees and hoverflies, were directly responsible for more than 20% of people’s vitamin A, folate, and vitamin E intake (Timberlake et al. 2026).

Large marine mammals can move nutrients upward in the water column by feeding at depth and releasing nutrient-rich waste nearer the surface, effectively recycling limiting elements back into the photic zone where primary production occurs (Roman and McCarthy 2010; Estes et al. 2016) and thereby supporting food webs that generate food for humans. Baleen whales (Fig. S1, no. 36), for example, can recycle substantial amounts of iron into marine food webs (Savoca et al. 2021).

Birds move nutrients over large scales, between continents and across the world. The flows associated with their seasonal migratory movements are substantial, given that these movements involve many billions of birds. Elements unavailable to humans can be transferred through birds as ecological vectors or mobile links. Their role in the cycling of basic elements influences levels of primary production and structures food webs that humans harvest and derive benefits from (Gaston 2022).

Seabirds operate as ecosystem engineers (Jones et al. 1994), linking nutrients from the sea, transferred through marine food webs into the zooplankton (Fig. S1, no. 15), fish and crustaceans that constitute their prey. Seabird guano is extremely rich in phosphorus, nitrogen, potash, and micronutrients (calcium, magnesium, sulphur, iron, manganese) and has been harvested from seabird colonies for at least 2000 years as a valuable fertiliser. Seabird guano transfers 10 000–100 000 tonnes of phosphorus to land annually, and waterfowl can input 40% of the nitrogen and 75% of the phosphorus entering a wetland (Sekercioglu 2006). The worldwide amounts of total nitrogen and total phosphorus excreted by seabirds are of similar magnitude to other sources considered in global cycles (Otero et al. 2018).

In Greenland, for example, 33 million pairs of breeding little auk *(Alle alle)* (a small alcid of ca 160 g, see Fig. S1, no. 35) have a profound impact on the composition of vegetation, by fertilising soils that are poor in nutrients (González-Bergonzoni et al. 2017). These seabirds deposit their guano far inland, significantly shaping the terrestrial ecosystem and associated human possibilities. The vegetation that their guano fertilises sustains hares, geese, foxes, reindeer (Fig. S1, no. 37), and muskoxen, all of which are important resources (food or clothing) for humans in the area (Mosbech et al. 2018).

Salmon (Fig. S1, no. 38) and other anadromous fish move marine-derived nutrients inland when they migrate upstream, where excretion and carcass decomposition fertilise rivers, riparian zones, and adjacent forests (Brandt et al. 2024). This pathway links ocean productivity to terrestrial nutrient availability: nutrients acquired at sea are re-released into freshwater and soil systems or accessed via predation, supporting food webs that humans also depend on.

Beyond transport, animals can alter elemental cycling by changing who eats whom, where, and how intensely. Predators can switch ecosystems between net carbon sources and sinks by reshaping herbivore behaviour and density; they also mediate carbon exchange and storage across landscapes (Carpenter et al. 2001; Schmitz et al. 2018). Large terrestrial herbivores add another mechanism: by grazing, trampling, and depositing dung and urine, they redistribute nutrients laterally across landscapes, concentrating and diluting fertility in distinct ways (e.g. Riesch et al. 2025).

Together, these examples illustrate how animal behaviour and life histories connect elemental and nutrient cycling across distinct and distant ecosystems—linking deep and surface waters, marine and freshwater, ocean and land, and nutrient-dense areas to broader landscapes. These ecological functionalities, of significance for the air we breathe, the water we drink, and the food we eat, can be lost even before the key species performing them are decimated or extinct (Díaz et al. 2020).

## Embodying the life-element interplay

In many parts of contemporary culture, humans and nature are still understood as separate realms. Frequently, the environment is treated as external to the economy and humans and our actions as external drivers of environmental change. Resources are extracted for human use, and nature is valued subject to human preferences. It has been suggested that this worldview and process of separation may have emerged with the Neolithic Revolution in the Middle East some 11 000 years ago, and was likely reinforced by monotheistic religions, modernity, technological progress, and economic development (White 1967; Loreau 2023).

Over recent decades, however, some fields have shifted their understanding of the human–environment interface from ‘nature for itself’, ‘nature despite people’, and ‘nature for people’ to an increasing recognition of the interplay of ‘people and nature’ and recently to ‘people with nature’ (Reyers and Bennett 2025). Such a shift reflects the emergent insight that human involvement with the biosphere is not simply of the mind, but of the whole body and its functions (including material, experiential, cognitive, emotional, and philosophical connections) interwoven with relations to other organisms in the making of the biosphere (e.g. Davidson-Hunt and Berkes 2003; Cooke et al. 2016; Ives et al. 2018). Our perspective has foregrounded and expanded on material embodiment—our bodies being sustained by life-mediated elemental flows—and, in doing so, has made visible why biosphere stewardship also requires relational embodiment: lived practices and institutions of care that sustain the relations enabling those essential flows (West et al. 2024).

Being ‘embodied’ in this way means that humans are tangibly immersed in the biosphere (Ingold 2011) and embedded in the world we try to understand (Folke et al. 2016; Haider and Rieser 2025). Our synthesis illustrates how human life has evolved in continuous interplay with the chemical elements available on Earth, the basic building blocks of life that continually flow through and form everything that is alive (Remick and Helmann 2023). Modern humans (*Homo sapiens*) evolved as part of the living planet, embedded in the biosphere, interwoven with the web of life and the basic chemical elements.

The basic elements move in biogeochemical cycles through life, the ocean and the atmosphere, and across land. Life, in its diversity, helps facilitate these flows in ways that both reflect and reshape the Earth system. Just as the human body relies on the circulation of oxygen, nutrients, and metabolic signals to sustain itself, so too does the Earth system rely on a global metabolism of chemical elements driven by biotic and abiotic processes.

The biosphere has existed for some 3.5 billion years. Modern humans appeared about 250 000 to 300 000 years ago (Bergström et al. 2021). If the history of *Homo sapiens* were compressed into a single day, humans lived as hunter-gatherers for 23 h of that day. Hunter-gatherers collected fruits, nuts, seeds, roots, leaves, eggs, insects, and caught and hunted fish, birds, and mammals, operating as part of food webs and participating in life’s mediation of the basic elements, adapting to environmental change, climate variability, and glacial-interglacial cycles (e.g. Dunne et al. 2016; Blockley et al. 2018; Bergström et al. 2021; Rathmann et al. 2024). Through their hunting, foraging, and burning, landscapes were transformed and megafauna extinct with cascading changes across many environments (Ellis 2015).

### Human reshuffling of the life-element interplay

The latest interglacial, Holocene, began 11 700 years ago. Here, climate variability was reduced and the environmental conditions for our species became more favourable, paving the way for the Neolithic Revolution, human settlements and civilisation. Human cultures started to shift from being participants to becoming a force in shaping the dynamics and mediation of global biogeochemical cycles (Turner et al. 1990; Vitousek et al. 1997; Steffen et al. 2004), and at an accelerating rate during the Industrial Revolution, placing humanity on the current trajectory of the Anthropocene (Steffen et al. 2007; Folke et al. 2021; Zalasiewicz et al. in press).

Consequently, processes mediating the chemical elements necessary for the functioning of the human body, which have predominantly been carried out by microorganisms, fungi, plants, and animals, have in Anthropocene times been substantially complemented, magnified, altered, and reshuffled by accelerating human actions and associated technologies. Climate change, eutrophication, atmospheric brown clouds, ocean acidification and deoxygenation, and salinisation of soils are examples of unwanted and unexpected side-effects of large-scale human-driven alteration of global biogeochemical cycles such as carbon, nitrogen, phosphorus, sulphur, and water. Humans are pushing such cycles outside the range of variability observed during the Holocene epoch (Richardson et al. 2023), with well-known worldwide impacts on human health and wellbeing (Rockström et al. 2023). For example, 80% of people in low-income countries live with degraded land, unhealthy air, and water stress (Damania et al. 2025).

In the Anthropocene, microbe-mineral interactions continue to shape the elemental flows, though now within a biosphere profoundly altered by human activities. Industrialisation, fossil-fuel combustion, mining, synthetic fertiliser use, and widespread antibiotic application are dramatically altering microbial communities both compositionally and functionally (Dong et al. 2022). Antibiotics, industrial chemistry, urban infrastructure, and intensified agriculture select for new microbial traits (including antimicrobial resistance) while also reducing everyday contact with diverse environmental microbiota (Gillings and Paulsen 2014; Bosch et al. 2024; Bradshaw 2024). These shifts show up both as microbial “abundance” (new genes and organisms thriving in human-shaped environments) and “absence” (diminished exposure to microbial diversity), with implications for inflammation, metabolism, and wellbeing (Yong 2016).

Excessive fertiliser use and monoculture farming have created widespread deficiencies of micronutrients such as iron, manganese, molybdenum, and zinc in soils, impacting soil health and reducing the nutritional content of food (De Groote et al. 2021; Lal 2024). Zinc scarcity in soil is an important constraint to crop production and the most widespread micronutrient deficiency in crops worldwide, aggravated by the progressive dilution of zinc in high-yield modern crops (Damania et al. 2025). Monoculture practices and use of pesticides change soil microbial communities, generally leading to reduced biodiversity, increased soil-borne diseases, and a shift towards fungal dominance (e.g. Belete and Yadete 2023; Liu et al. 2023). Pesticide use in woodlands, grasslands and croplands causes complex and widespread non-target effects on soil biodiversity, including changes in microbial functions like phosphorus and nitrogen cycling, and suppresses beneficial taxa, such as mycorrhizal fungi and bacterivore nematodes (Köninger et al. 2026).

The pervasive human-driven decline of biological populations and diversity, alongside the loss of species and their functions (Estes et al. 2011; Díaz et al. 2019) as well as introductions of invasive species, have significantly affected the relations of life and the elemental cycles. It has been estimated that fish biomass and cycling rates in the ocean have been reduced by nearly half, reflecting a major biogeochemical impact of fisheries (Bianchi et al. 2021). Simplified food webs in ecosystems such as lakes, coral reefs, grasslands, or forests make them vulnerable to changes and disturbances that previously could be absorbed (e.g. Bellwood et al. 2004; Levia et al. 2020). Such erosion of resilience has caused tipping points and regime shifts with loss of essential ecosystem services (Folke et al. 2004; Rocha et al. 2018).

The simplification of landscapes and seascapes and an increasingly urbanised planet have considerably altered such life-element dynamics, challenging biosphere resilience (Seto et al. 2011; Nyström et al. 2019). Evidence grows that contemporary built environments alter microbiome exposures and reduce the microbial diversity essential for human health and wellbeing (Bosch et al. 2024).

The development and use of novel entities—that is, engineered materials or organisms not previously known to the Earth system, such as synthetic chemicals, plastics, modified life forms and nanomaterials, as well as elements mobilised by anthropogenic activities, such as heavy metals—substantially influence the biosphere’s life–element dynamics, with consequences for human health and wellbeing (Persson et al. 2022). Examples include the accumulation of plastics and PFAS in the human body and chemicals and heavy metals in breast milk (Lewis et al. 2015; Krasucka et al. 2022; Serreau et al. 2024).

It remains to be seen in what ways the geohistorically novel configuration of the Anthropocene biosphere and the recent and widespread reshuffling of life-element relations and addition of novel entities will continue to feed back on human life and wellbeing.

### Independent or inseparable?

The technosphere plays a significant role in the dynamics of the contemporary Earth system and supports humans in numerous important ways, including the provision of food, shelter, transportation, long-distance communication, and material wealth (Galbraith et al. 2025). In 2020, the ‘technomass’ exceeded the dry weight of the biomass of all living organisms on Earth (Elhacham et al. 2020).

Has the pervasive technological development, combined with the accelerating economic, social and cultural changes of recent history (e.g. McNeill 2000; Zalasiewicz et al. 2017; Renn 2022) separated the human body and its life processes from dependence on life-mediated flows of the essential chemical elements of planet Earth? The answer is no.

Although humanity has become a major driving force altering the flows of Earth’s chemical elements, in patterns, magnitude and scale, and reshuffles critical life-element relations in new ways, this does not imply independence of human beings from fundamental life-element processes and relations.

Furthermore, although many relations of the complex life-element interplay have been substantially disrupted by humankind, for example through simplification, loss of trophic relations and functional diversity, this does not imply independence either. On the contrary, intertwined life-element relations, albeit extensively modified in their interplay and dynamics, and in some cases reconfigured in novel ways, remain fundamental for us humans, simply because, as shown in the article, we humans are not external to, but embodied and intertwined beings of the biosphere.

Humans have truly become the dominant life form in the biosphere, a key player in the relations and functioning of this complex, adaptive and evolving Earth system. As interdependent components of these dynamics, humans, the web of life and the basic elements are inseparable.

Attending to the profound material inseparability of life-element relationships raises important questions about the conditions required for nurturing life in the Anthropocene and the consequences of disrupting the biogeochemical foundations upon which all living systems, including our own, depend.

### Stewardship of life-element mediation

Because human bodies depend on life-mediated elemental flows, biosphere stewardship is not only about nature protection and environmental management in general; it is also about sustaining the living relations that make human existence materially possible. This fundamental inseparability calls for active stewardship of life–element mediation processes and relations. It means appreciating, regenerating, and revitalising relations that sustain rather than degrade the life-enabling movement of elements through Earth’s living tissues—microbial, fungal, plant, animal, and human—across scales (Fig. 1). It also means recognising that our relations with the biosphere emerge through ongoing interactions among environment, body and mind over time (Cooke et al. 2016).

Building on stewardship scholarship, this implies not only an ethic of care but also the mobilisation of knowledge and agency through concrete actions, practices, and institutions that safeguard, navigate and responsibly shape the complex living relations on which human wellbeing depends (e.g. Berkes and Folke 1998; Olsson et al. 2004). It entails valuing the competencies and skills embedded in stewardship practices and recognising the making of meaning, respect and dignity associated with them (e.g. Nabhan 1997; Bennett et al. 2018). It also involves cherishing stewardship as an ongoing learning process that builds resilience capacities for dealing with complexity, uncertainty and change (e.g. Fischer et al. 2026).

A rapidly developing scientific and practical knowledge base now addresses the benefits of nurturing processes and relationships among humans, the wider biosphere, and the cycles of chemical elements. This practical orientation is visible across several domains, although usually under separate labels: microbiome health, nature exposure, soil health, agroecology, rewilding, aquatic food systems, and nature-based solutions.

In public health and clinical research, growing attention is being paid to gut microbiome health and diversity alongside dietary and environmental contexts. Relevant lines of inquiry range from the “biodiversity hypothesis” concerning allergic disease (Haahtela 2019; Roslund et al. 2022) to the gut–brain axis as a channel linking microbial ecologies to stress and mental health (Mayer et al. 2022), with microbiome-based therapies entering formal clinical guidance (Peery et al. 2024; Manghi et al. 2026).

Work on nature exposure and mental health likewise indicates that biodiversity and lived environments can shape physiological regulation through multiple pathways (Bratman et al. 2015; Bratman et al. 2019; van den Bosch and Meyer-Lindenberg 2019).

Agricultural practices in Europe are increasingly integrating soil health management measures that actively cultivate microbial relationships (Reese 2025), echoing broader efforts to operationalise agroecological principles in policy and practice (FAO 2018). Such soil–root–microbiome practices in regenerative agriculture (e.g. Mooney et al. 2024) can also help revitalise degraded land and improve water retention and quality, carbon storage, biodiversity and pest management (Beillouin et al. 2021; Coban et al. 2022).

Other examples gaining attention include landscape rewilding, grazing management for soil health and biodiversity, marine protected areas that can support seafood production, and, more broadly, the application of nature-based approaches to regenerative and healthy landscapes, with social-ecological systems understood as intertwined and co-dependent (e.g. Teague and Kreuter 2020; Carver et al. 2021; Sala et al. 2021; Welden et al. 2021; Seddon 2022).

Across these domains, a common feature is that they work with living mediators of elemental flow rather than treating food, water, health and biodiversity as separate concerns. Such practices, with examples presented in the Supplementary information, reflect a shift from managing isolated resources or environmental outcomes towards caring for mediation processes: microbial metabolism, soil–root–fungal exchange, animal movement, trophic relations and hydrological regulation, while limiting disruptions to elemental flows caused by novel entities.

These are illustrative examples of efforts towards biosphere stewardship (Folke et al. 2016; Chapin et al. 2022). They can be considered efforts that make intertwined co-dependence in the Anthropocene biosphere tangible by reimagining relations and acting on them to regenerate and revitalise life-sustaining flows.

In Box 1 we propose six guiding principles for redirecting human action in the Anthropocene towards revitalising life–element processes and relations and strengthening the long-term resilience of our living planet (Søgaard Jørgensen et al. 2024). The recognition, appreciation and engagement of societies and cultures with the inseparable life-element interplay are fundamental for progress towards sustainable futures.

**Box 1:** Guiding principles for revitalising life–element relations

**Shift perspectives and action from viewing bacteria primarily as pathogens towards promicrobial governance of metaorganismic relations**. This includes monitoring and supporting host–microbial symbioses in bodies, homes, healthcare settings and wider environments^a^	
**Reverse nutrient dilution in the Anthropocene through regenerative agriculture that enriches soil biota**. Practices that restore soil biota and root–microbial–fungal relations can improve nutrient density, water retention, biodiversity and resilience^b^	
**Work with, rather than bypass, the living mediators that sustain elemental flows and the quality of air, water and food**. These include soil microbiomes, mycorrhizal fungi, marine food webs, mobile link species and animal-mediated nutrient transport^c^	
**Redirect urban development to expand access to biodiverse green space and contact with nature**. Such environments can support immune function, mental health and more diverse human–microbial exposure^d^	
**Foster relational approaches in science, technology, practice and governance**. Such approaches better emphasise that being in the biosphere emerges through enacting humility, wonder, care and reciprocity for the continually unfolding relations that mobilise and circulate life-sustaining essential elements^e^	
**Appreciate our deep evolutionary inseparability from the biosphere as precondition for health and existence**. Human wellbeing depends on life-mediated elemental relations that long predate our species and continue to sustain human life^f^	

^a^e.g. Bosch and McFall-Ngai (2011), Søgaard Jørgensen et al. (2017), Sharma et al. (2021)

^b^e.g. Coban et al. (2022), Raaijmakers and Kiers (2022), Mooney et al. (2024)

^c^e.g. Schmitz et al. (2018), Crowther et al. (2019), Hicks et al. (2019)

^d^e.g. Bratman et al. (2019), van den Bosch and Meyer-Lindenberg (2019), Bosch et al. (2024)

^e^e.g. Cooke et al. (2016), West et al. (2020), Smith et al. (2026)

^f^e.g. Latour and Weibel (2020), Folke et al. (2021), Kaspari (2021)

## Concluding remarks

In this article we position the human body as an open, dynamic system, embedded in and sustained by the broader life-metabolism of the biosphere. The synthesis illustrates that the biosphere is not only around us but also moves through us, that we humans are open systems, intertwined beings in continuous exchange with life via chemical elements, that we embody life-sustaining relationships and processes of the biosphere.

Our bodies have evolved with, and are fundamentally dependent on and inseparable from, the essential elements required to operate and function. Biospheric processes like photosynthesis for oxygen and carbohydrates, rainfall mediated by trees sustaining soil moisture, mineralisation by microorganisms in soil for plant growth, or ions and omega-3 s accumulated and synthesised in marine food webs remain of significance for our existence. They demonstrate the importance of our entanglement with the biosphere for health and wellbeing, throughout human history and into the future.

Hence, we humans are not external drivers acting on the Earth system but physically and materially interwoven and immersed within the biosphere. Life keeps us alive. This reality makes people embodied participants in shaping the biosphere, consciously or unconsciously.

The empirical synthesis provides a material and biogeochemical grounding for rethinking worldviews and values that currently treat the planet as a backdrop to where we live, a surface we inhabit, a resource to be extracted, or an externality that we impact. In the Anthropocene trajectory, we humans have become a global force shaping and challenging our own intertwined future and the future of civilisation on the living planet.

Fortunately, there are encouraging signs of reconnection in mindsets, worldviews, and actions towards cultural appreciation of humans as beings in the biosphere and the beauty and wonder of being part of it. Here, we have clarified that such an appreciation is not simply a value proposition but a biogeochemical reality: human existence depends on life-mediated elemental flows. This reality persists continuously, day after day, year after year, also in the Anthropocene. Reconnecting therefore entails acting upon the understanding that humans are intertwined beings embodying the biosphere. Stewardship grounded in knowledge, skills and practices that foster appreciation of and care for the living relations that sustain those flows becomes of strategic importance for development and wellbeing.

## Supplementary Information

Below is the link to the electronic supplementary material.

Supplementary file1 (PDF 1279 KB)
