# Supplementary material for: Humans: Intertwined with life and the basic elements in the Anthropocene biosphere
Source: Ambio. 2026 Sep 4;55(10):2295–320. doi: 10.1007/s13280-026-02474-z (PMC13554003; doi:10.1007/s13280-026-02474-z)
Supplement: Supplementary file 1 [file 13280_2026_2474_MOESM1_ESM.pdf]

Supplementary Information

This Supplementary Information has not been peer reviewed.

Title: Humans - Intertwined with Life and the Basic Elements in the Anthropocene Biosphere

Supplementary Note S1. Annotated version of Fig. 1: The Elements of Life

The Elements of Life (Fig. 1), ink drawing and aquarelle on watercolour paper by Tone Bjordam (2026), is an interpretive synthesis of life-mediated elemental flows illustrating that *humans are intertwined beings embodying the biosphere*. Its cyclic composition situates a human figure within, rather than outside, the biosphere, linking processes in the human body to those operating through soil, water and air. The large bottommost elemental, crystalline and mineral forms are inspired by cordierite and represent the geochemical foundations of life. Throughout the composition, roots, mycelia, soil organisms, plants, pollinators, aquatic microorganisms, fish, birds and mammals appear alongside elemental, mineral and crystalline forms. The motifs are inspired by Scandinavian temperate ecosystems.

In this Supplementary Note, numbers 1–58 correspond to the components of the annotated version of the artwork in Fig. S1. The numbers identify individual components; they do not represent a temporal sequence or a prescribed pathway. The cyclic composition reflects reciprocal and multidirectional relations among life, elements and environments.

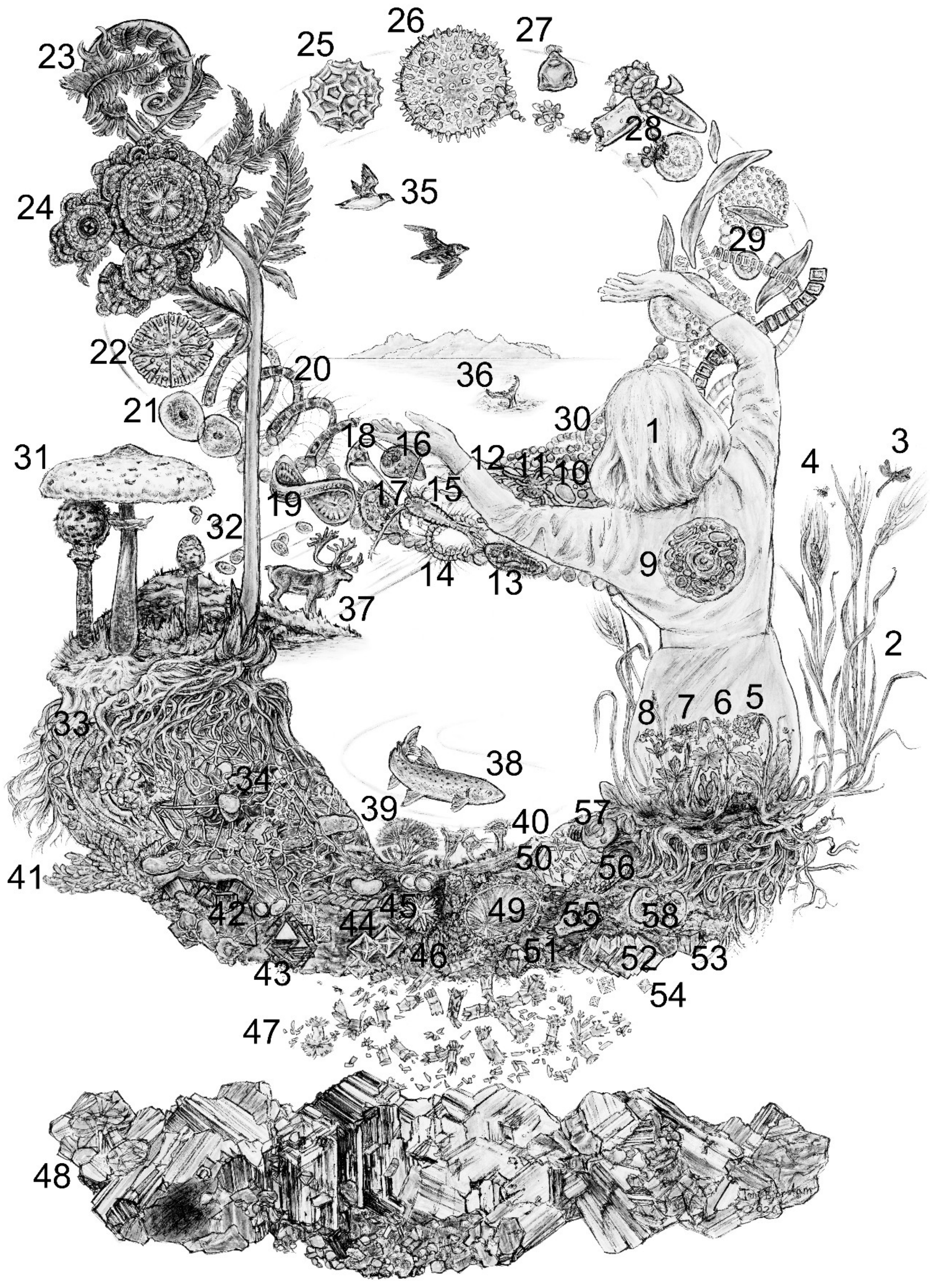

Figure S1. Annotated version of *The Elements of Life*, ink drawing on watercolour paper, Tone Bjordam, 2026: an interpretive synthesis of life-mediated elemental flows illustrating that *humans are intertwined beings embodying the biosphere*. Numerals locate the motifs described in Supplementary Note S1, they do not indicate a sequence or a single pathway.

1. Human figure is Anna Asplind from Asplind and Bjordam's project In Sync (2025), in which the artists explored movements and formations in the surrounding landscape.
2. Barley (*Hordeum* sp.). Barley plants form part of the vegetation surrounding the human figure.
3. Damselfly ('the beautiful demoiselle'; *Calopteryx virgo*). A damselfly placed among the meadow vegetation around the human figure.
4. Bumblebee (*Bombus* sp.). A pollinator shown above the barley.
5. Cowslip primrose (*Primula veris*). One of the wild meadow flowers surrounding the human figure.
6. Bush vetch (*Vicia sepium*). A further meadow plant shown near the base of the human figure.
7. Lady's mantle (*Alchemilla vulgaris*). A further meadow plant shown near the base of the human figure.
8. Wild strawberry (*Fragaria vesca*). A further meadow plant shown near the base of the human figure.
9. Human cell. A human cell represented on the figure's back, linking the human body to the microscopic scales explored in the artwork.
10. Alphaproteobacterium (class Alphaproteobacteria). A bacterial motif positioned in front of the human figure.
11. Asgard archaeon ('*Candidatus Lokiarchaeum ossiferum*'). An Asgard archaeon near the human figure, included as a motif of deep evolutionary connection between microbes and complex life.
12. Deltaproteobacterium. A bacterial motif positioned alongside the Asgard archaeon and alphaproteobacterium, evoking proposed syntrophic associations among archaea and bacteria in models of early eukaryotic evolution.
13. *Paramecium* (*Paramecium* sp.). A ciliated protist situated below the human figure's arm.
14. Horseshoe-shaped diatom (*Chaetoceros debilis*). A phytoplanktonic diatom represented in a curved, horseshoe-like form.
15. Calanoid copepods (order Calanoida). Yellow, orange and blue zooplankton forms within the aquatic part of the composition.
16. Green ciliate protist (*Stentor coeruleus*). A green, trumpet-shaped ciliate included among the aquatic microorganisms.
17. Pink and blue stentors (*Stentor* spp.). Additional trumpet-shaped ciliate forms included alongside the green *Stentor* motif.
18. Dinoflagellate (*Tripos muelleri*).
19. Figure-of-eight–like diatom form (*Surirella*). A twisted, looping diatom silhouette represented in the water portion of the composition.
20. Spiral diatom form (again *Chaetoceros debilis*). A separate spiral diatom motif.
21. Cyanobacteria (*Prochlorococcus marinus*). Two green cyanobacterial forms representing the abundant marine photosynthesiser.
22. Green alga (*Micrasterias*). A round green alga.
23. Ostrich fern (*Matteuccia struthiopteris*). A fern drawn from plants observed in the forest. Its emerging shoots are edible when cooked and are rich in Vitamin C.
24. Vitamin C (ascorbic acid). A crystal-like microscopic pattern positioned on top of the ostrich fern.
25. Gazania pollen (treasure flower, *Gazania* sp.). One of three enlarged pollen grains shown in the aerial part of the composition.
26. Pumpkin pollen (*Cucurbita* sp.). One of three enlarged pollen grains shown in the aerial part of the composition.
27. Birch pollen (*Betula* sp.). A pink pollen grain based on a stained micrograph. The stain highlights the exine, the resistant outer wall of pollen grains.
28. Airborne particles and dust. An assemblage of small particles in the aerial part of the composition, evoking the transport of mineral, organic and biological material through air.

29. Phytoplankton motif. A separate visual motif inspired by a photograph of marine phytoplankton. Named diatoms and cyanobacteria are identified elsewhere in this guide.
30. Chains of cyanobacteria. Chain-forming cyanobacterial cells shown in the water and air portion of the composition.
31. Parasol mushroom (*Macrolepiota procera*). A large parasol mushroom, with mycelium extending into the soil.
32. Parasol-mushroom spores. Enlarged spores of *Macrolepiota procera* in the air beneath the mushroom.
33. Nematode. An unidentified soil nematode represented among the soil biota.
34. Soil bacteria. Bacterial forms interwoven with plant roots and fungal hyphae in the soil. The motif evokes the root–soil–microbial relations through which organic matter and mineral forms are transformed and made available to plants.
35. Little auk (*Alle alle*). Two seabirds shown in flight, linking marine food webs to avian movement across sea and land.
36. Humpback whale (*Megaptera novaeangliae*). The fluke of a diving marine mammal in the waters surrounding the island of Landegode, near Bodø, in the north of Norway. The motif represents animals that mediate nutrient transport and elemental cycling within ocean ecosystems.
37. Reindeer (*Rangifer tarandus*). A terrestrial mammal motif.
38. Atlantic salmon (*Salmo salar*). A salmon in the water, representing fish as both participants in marine food webs and mobile links between aquatic and terrestrial systems.
39. Fan-shaped diatom (*Licmophora flabellata*). A marine diatom represented by its fan-shaped form.
40. Additional stentors (*Stentor* spp.). Three further trumpet-shaped ciliate protists depicted in the water.
41. Copper oxide crystals. Crystalline copper-oxide forms represented under microscopy, illustrating one way copper occurs in mineral contexts.
42. Chalcopyrite and pyrite. A chalcopyrite motif with pyrite crystals above it, together with a second chalcopyrite form. Chalcopyrite primarily contains copper, iron and sulphur, sometimes with trace elements like cobalt, nickel, indium, thallium, selenium, tellurium, silver and gold.
43. Sphalerite. Black tetrahedral crystals of sphalerite, a zinc sulphide mineral.
44. Calcium crystals. Microscopy-inspired crystalline calcium forms.
45. Zinc oxide crystals. Star-shaped zinc oxide crystals represented through scanning-electron-microscopy-inspired imagery.
46. Magnesium-containing mineral motif. A microscopy-inspired crystalline form, drawing on blue brucite and associated carbonate minerals. The motif evokes magnesium in mineral contexts rather than depicting a single identified mineral specimen.
47. Magnesium sulphate crystals. A crystal form shown under polarised-light microscopy.
48. Cordierite-inspired mineral assemblage. The large bottommost motif is inspired by the mineral composition and microscopic appearance of cordierite. It is an interpretive mineral assemblage rather than a literal specimen.
49. Crystallised sulphur. A colourful fan-like crystalline sulphur motif within the soil.
50. Calcium carbonate (calcite) crystals and microbially induced carbonate precipitation. A calcite-crystal motif informed by microbially induced carbonate precipitation, through which microbial activity can promote the precipitation and growth of calcium carbonate in soils and engineered settings (Shivaprakash and Burns 2025).
51. Hematite. A hematite crystal, representing an iron oxide mineral.
52. Halite. A halite crystal, commonly known as rock salt.
53. Blue halite. A blue visual variant of halite.
54. Salt crystals (sodium chloride). A separate drizzling salt-crystal motif, chemically equivalent to halite but visually distinct in the artwork.
55. Green testate amoeba. A testate amoeba shown in the soil.

56. Transparent testate amoeba (*Euglypha tuberculata*). A transparent testate amoeba represented from a micrograph.
57. Golden bowl-shaped testate amoeba (*Arcella* sp.). A testate amoeba associated with freshwater, moss and soil environments.
58. Manganese-oxidising bacteria. A bacterial motif informed by microorganisms that can catalyse the oxidation of Mn(II) to manganese oxides, illustrating microbial mediation of elemental form and mobility (Lu et al. 2021).

S1.1. Acknowledgement note

The Elements of Life is an original artwork by Tone Bjordam. In developing the composition, the artist drew on direct observation, her own photographs and a range of publicly accessible visual materials, including photomicrographs, natural-history photography, mineral imagery, scientific figures and institutional educational resources. These materials informed the interpretive rendering of individual motifs across the artwork. They are acknowledged here as sources of visual and contextual inspiration.

Supplementary Note S2: Oxygen, iron and bacteria

S2.1. Bacteria, oxygen, and body functions

Cyanobacteria evolved at least 2.4 billion years ago. They were among Earth's earliest known oxygenic photosynthesisers, making food using water and the Sun's energy (Sánchez-Baracaldo and Cardona 2020). The microbial processes were potentially facilitated by manganese oxide minerals that may have catalysed light-driven water-splitting reactions and led to the release of molecular oxygen (O_2) (Lu et al. 2021). Over time, more oxygen was produced than could be absorbed, eventually leading to the accumulation of oxygen in the atmosphere, a process known as the Great Oxidation Event. This catalysed a dramatic rise in oxygen, making the environment less hospitable for the anaerobic bacteria, since such bacteria could not tolerate oxygen. The door thereby opened for the evolution of fast-growing bacteria able to use oxygen to break down sugar such as glucose and other nutrients to produce a large amount of energy in the form of ATP, the main energy-carrying molecule in human cells that powers biochemical reactions.

Simultaneously, microbial transformation of iron minerals helped regulate reactive oxygen species in increasingly oxygenated environments (Swanner et al. 2015), while shifts in mineral weathering increased the bioavailability of key metal cofactors and nutrients, including copper and phosphorus, supporting the evolution of metabolic systems (Hazen et al. 2008).

The accumulation of oxygen was associated with the evolution of free-living eukaryotic cells such as amoebae, which could use oxygen to generate energy from engulfed (phagocytosed) bacteria. Vertebrate macrophages, including those of humans, retain phagocytic and killing mechanisms with deep evolutionary roots in amoeboid unicellular eukaryotes. The basic cellular machinery for engulfing and digesting particles, like lysosomes and the use of oxidants, is shared between modern amoebae and macrophages, suggesting an ancient common origin for this defensive function (Gaudet et al. 2016).

Oxygen can be toxic. Its metabolism can lead to the formation of highly reactive free radicals (ROS), such as the superoxide anion ($O_2^{\bullet-}$). These free radicals can start chain reactions, which damage cell membranes and other important cellular components. Therefore, many organisms that live in the presence of oxygen have evolved antioxidant systems such as superoxide dismutase (SOD) that neutralise ROS. SODs work by catalysing the conversion of the harmful superoxide radical into hydrogen peroxide and oxygen. The resulting hydrogen peroxide is then controlled by other antioxidant systems. This process uses redox-active metal ions like copper, zinc, or manganese in the enzyme's active site, which cycles between

oxidation and reduction states to effectively neutralise the superoxide (Wang et al. 2018).

S2.2. Iron and bacteria in our body

Cells in our body are dependent on oxygen. Iron fulfils a central function in transporting oxygen in our body by being present in the red blood cells and binding oxygen without itself being oxidised and thereby delivering oxygen to various cells in the body, where it is used by mitochondria in aerobic respiration to generate energy.

Iron is also essential for the bacteria in our body. They usually capture iron by releasing iron-binding molecules, so-called siderophores. Siderophores have a high affinity for iron and are crucial for microorganisms that have limited access to it, which is common in aerobic environments where iron is often insoluble. In pathogenic microorganisms, siderophores are a key virulence factor, as they enable the microbes to acquire iron from their host.

Neutrophils, the most common type of white blood cell, act as the first line of defence for the immune system to fight infections, particularly bacterial ones. They protect our body surfaces, such as the nose, by migrating to sites of inflammation. There they engulf (phagocytose) bacteria and then use enzymes, antimicrobial peptides, and ROS to kill the pathogen. There are also small molecules (antioxidants) that can neutralise ROS. Some we can make ourselves in the liver, and some we get from food, such as vitamin C.

Although iron is critical for host cellular and immune function, it can also promote the growth of pathogenic microbes, such as *Staphylococcus aureus* (Zhao et al. 2024). In the nose the availability of nutrients is scarce, preventing excessive growth of pathogenic bacteria. During inflammation in the airways more nutrients become available promoting the growth of pathogenic microbes, which can also withstand phagocytosis by infiltrating neutrophils. However, growth of the pathogen also results in increased bacterial production of ROS, which can become toxic for the pathogen unless antioxidants are provided by the host.

Under iron-restricted conditions, siderophores become contested resources among microbial taxa. In the nasal microbiome, for example, *S. aureus* relies heavily on siderophore-mediated iron acquisition for successful colonisation (Stubbendieck et al. 2019). However, evidence indicates that commensal *Corynebacterium* species can suppress *S. aureus* growth by consuming staphylococcal siderophores to access nutritional iron, often without incurring the energetic cost of producing siderophores themselves (Zhao et al. 2024). This competitive exploitation of pathogen-derived siderophores by commensal microbes protects against pathogen proliferation and illustrates another mechanism through which microbial mediation of key nutritional elements supports host physiological health.

Supplementary Note S3: Illustrative stewardship practices for life-mediated elemental relations

Human lives depend on living processes that mobilise, transform, transport, incorporate and recycle elements. Stewardship can therefore involve more than conserving nature in general: it can protect, restore or govern the relationships through which life makes essential elements available, moves them between environments, and limits harmful disruption.

The examples below illustrate such work across everyday environments, agriculture, water provision, food systems, ecological restoration, chemical governance and Indigenous stewardship. They are not intended as a systematic review or as universally transferable models. Each depends on particular ecological conditions, institutions, forms of knowledge and relations of care.

S3.1. Biodiverse living environments and microbial relations

The Finnish daycare biodiversity intervention illustrates how everyday built environments can be redesigned to support contact with diverse environmental microbiota. Urban daycare yards were enriched with forest floor, sod and gardening materials. Over the intervention period, these changes were accompanied by changes in children's environmental, skin and gut microbiota and in markers associated with immune regulation (Roslund et al. 2020). The example shows one way stewardship can work through educational settings in which children encounter soils, plants and microbial life.

S3.2. Regenerating soil–root–fungal relations

Agricultural stewardship can seek to work with, rather than bypass, the biological processes through which soils retain, release and cycle nutrients. Andhra Pradesh Community-Managed Natural Farming provides a large-scale example of efforts to reduce dependence on external inputs and strengthen soil-centred farming practices (Duddigan et al. 2023; Jaacks et al. 2023). Evidence from other contexts suggests that, when compared to conventional farming, regenerative practices can be associated with stronger soil-health indicators and differences in crop nutrient profiles, although these findings remain context dependent (Montgomery et al. 2022). Experimental research further indicates that soil biota can enhance crop nutrient uptake and reduce nutrient leaching (Bender and van der Heijden 2015).

S3.3. Water systems designed around ecological mediation

Riverbank filtration uses the biological, chemical and physical processes of riverbeds and aquifers as part of drinking-water treatment. As water moves through sediments, filtration, sorption and microbial degradation can reduce pathogens and a range of organic and inorganic contaminants before abstraction. Its effectiveness depends on local hydrogeology, contaminant type and operational design, but it offers a clear example of water provision that retains ecological mediation as part of the treatment process (Uwimpaye et al. 2025).

S3.4. Aquatic food systems and micronutrients

Nutrition-sensitive carp–mola polyculture in Bangladesh illustrates food-system design oriented towards micronutrient availability rather than primarily to biomass production. The approach combines carp with mola (*Amblypharyngodon mola*), a small indigenous fish rich in vitamin A, calcium and other micronutrients. Roos et al. (2007) found that including mola did not reduce carp production while creating potential nutritional benefits. More broadly, the Blue Food Assessment shows why aquatic foods should not be treated simply as sources of protein: their nutritional diversity can be central to reducing micronutrient deficiencies (Golden et al. 2021).

S3.5. Restoring animal-mediated nutrient transport

Seabird-island restoration shows how stewardship can repair disrupted nutrient transport across ecological settings. Removing invasive rats can allow seabird colonies to recover, restoring the transfer of marine-derived nutrients to islands through guano. Research from rat-free islands links higher seabird abundance and nutrient deposition to stronger reef productivity, ecosystem functioning and resilience (Graham et al. 2018; Benkwitt et al. 2023). As a result, species conservation efforts are also seen to restore animal-mediated elemental relations connecting ocean, land and reef ecosystems.

S3.6. Reducing novel-entity disruption

Governance of chemicals and waste can help limit disruptions to microbial, physiological and ecosystem processes. The Global Framework on Chemicals, adopted at the Fifth International Conference on Chemicals Management in 2023, provides an international pathway for action across the life cycle of chemicals, products and waste. It includes a target to discontinue highly hazardous pesticides in agriculture by 2035 and to support transition to

safer alternatives (UNEP 2023). Pesticide pollution can disrupt plant-beneficial soil bacteria (Qiu et al. 2025). Evidence on pesticide-associated changes to the gut microbiota and microbiota–gut–brain axis remains evolving and should be interpreted cautiously (Matsuzaki et al. 2023).

S3.7. Situated relational stewardship

Indigenous cultural burning provides an example of stewardship enacted through long-standing place-based knowledge, care and authority. Research developed with Karuk practitioners has documented the ecological effects of cultural burning, including changes in vegetation and soil properties, while demonstrating the value of bringing Indigenous and western scientific approaches into dialogue (Greenler et al. 2024; Mariani et al. 2024). Its relevance here is not that cultural burning offers a universally applicable technique, but that it shows stewardship as a situated practice of maintaining relationships among fire, soils, plants, animals and people.

Table S2. Illustrative stewardship pathways, practices and life-mediated relations addressed.

Pathway	Implementation example	Supporting mechanism/framing
Human microbiome / skin	Finnish daycare biodiversity intervention	Environmental microbiota, skin/gut microbiota, immune regulation
Soil–root–fungal relations	APCNF / Regenerative agriculture	Soil-health indicators; context-dependent crop nutrient profiles; soil biota and arbuscular mycorrhizal fungal contributions to nutrient uptake.
Water systems	Riverbank filtration	Microbial/biogeochemical filtration of contaminants
Marine/aquatic food systems	Carp–mola polyculture	Blue Food Assessment
Animal-mediated nutrient transport	Seabird island restoration	Marine-derived nitrogen and phosphorus transfer through seabird guano; consequences for reef productivity, functioning and resilience.
Novel entities	Global Framework on Chemicals	Reducing chemical pressures on plant-beneficial soil bacteria; evidence on gut-microbiota disruption.
Situated relational stewardship	Indigenous cultural burning	Practices of care, knowledge and agency; soil carbon/nitrogen/fire-mediated relations

References

Bender, S.F., and M.G.A. van der Heijden. 2015. Soil biota enhance agricultural sustainability by improving crop yield, nutrient uptake and reducing nitrogen leaching losses. *Journal of Applied Ecology* 52: 228–239. https://doi.org/10.1111/1365-2664.12351

Benkwitt, C.E., C. D'Angelo, R.E. Dunn, R.L. Gunn, S. Healing, M.L. Mardones, J. Wiedenmann, S.K. Wilson, et al. 2023. Seabirds boost coral reef resilience. *Science Advances* 9: eadj0390. https://doi.org/10.1126/sciadv.adj0390

Duddigan, S., L.J. Shaw, T. Sizmur, D. Gogu, Z. Hussain, K. Jirra, H. Kaliki, R. Sanka, et al. 2023. Natural farming improves crop yield in SE India when compared to conventional or organic systems by enhancing soil quality. *Agronomy for Sustainable Development* 43: 31. https://doi.org/10.1007/s13593-023-00884-x

Gaudet, R.G., C.J. Bradfield, and J.D. MacMicking. 2016. Evolution of Cell-Autonomous Effector Mechanisms in Macrophages versus Non-Immune Cells. *Microbiology Spectrum* 4. https://doi.org/10.1128/microbiolspec.mchd-0050-2016
Golden, C.D., J.Z. Koehn, A. Shepon, S. Passarelli, C.M. Free, D.F. Viana, H. Matthey, J.G. Eurich, et al. 2021. Aquatic foods to nourish nations. *Nature* 598: 315–320. https://doi.org/10.1038/s41586-021-03917-1
Graham, N.A.J., S.K. Wilson, P. Carr, A.S. Hoey, S. Jennings, and M.A. MacNeil. 2018. Seabirds enhance coral reef productivity and functioning in the absence of invasive rats. *Nature* 559: 250–253. https://doi.org/10.1038/s41586-018-0202-3
Greenler, S.M., F.K. Lake, W. Tripp, K. McCovey, A. Tripp, L.G. Hillman, C.J. Dunn, S.J. Prichard, et al. 2024. Blending Indigenous and western science: Quantifying cultural burning impacts in Karuk Aboriginal Territory. *Ecological Applications* 34: e2973. https://doi.org/10.1002/eap.2973
Hazen, R.M., D. Papineau, W. Bleeker, R.T. Downs, J.M. Ferry, T.J. McCoy, D.A. Sverjensky, and H. Yang. 2008. Mineral evolution. *American Mineralogist* 93: 1693–1720. https://doi.org/10.2138/am.2008.2955
Jaacks, L.M., L. Bliznashka, P. Craig, M. Eddleston, A. Gathorne-Hardy, R. Kumar, S. Mohan, J. Norrie, et al. 2023. Co-Benefits of Largescale Organic farming On huMan health (BLOOM): Protocol for a cluster-randomised controlled evaluation of the Andhra Pradesh Community-managed Natural Farming programme in India. *PLOS ONE* 18: e0281677. https://doi.org/10.1371/journal.pone.0281677
Lu, A., Yan Li, F. Liu, Y. Liu, H. Ye, Z. Zhuang, Yanzhang Li, H. Ding, et al. 2021. The photogeochemical cycle of Mn oxides on the Earth's surface. *Mineralogical Magazine* 85: 22–38. https://doi.org/10.1180/mgm.2021.10
Mariani, M., A. Wills, A. Herbert, M. Adeleye, S.A. Florin, H. Cadd, S. Connor, P. Kershaw, et al. 2024. Shrub cover declined as Indigenous populations expanded across southeast Australia. *Science* 386: 567–573. https://doi.org/10.1126/science.adn8668
Matsuzaki, R., E. Gunnigle, V. Geissen, G. Clarke, J. Nagpal, and J.F. Cryan. 2023. Pesticide exposure and the microbiota-gut-brain axis. *The ISME Journal* 17: 1153–1166. https://doi.org/10.1038/s41396-023-01450-9
Montgomery, D.R., A. Biklé, R. Archuleta, P. Brown, and J. Jordan. 2022. Soil health and nutrient density: preliminary comparison of regenerative and conventional farming. *PeerJ* 10: e12848. https://doi.org/10.7717/peerj.12848
Qiu, D., Y. Wang, N. Xu, B. Chen, Y. Zhu, Z. Zhang, Q. Zhang, T. Lu, et al. 2025. Global variation in plant-beneficial bacteria in soil under pesticide stress. *Nature Communications* 16: 10685. https://doi.org/10.1038/s41467-025-65719-7
Roos, N., Md.A. Wahab, M.A.R. Hossain, and S.H. Thilsted. 2007. Linking Human Nutrition and Fisheries: Incorporating Micronutrient-Dense, Small Indigenous Fish Species in Carp Polyculture Production in Bangladesh. *Food and Nutrition Bulletin* 28: S280–S293. https://doi.org/10.1177/15648265070282S207
Roslund, M.I., R. Puhakka, M. Grönroos, N. Nurminen, S. Oikarinen, A.M. Gazali, O. Cinek, L. Kramná, et al. 2020. Biodiversity intervention enhances immune regulation and health-associated commensal microbiota among daycare children. *Science Advances* 6: eaba2578. https://doi.org/10.1126/sciadv.aba2578
Sánchez-Baracaldo, P., and T. Cardona. 2020. On the origin of oxygenic photosynthesis and Cyanobacteria. *New Phytologist* 225: 1440–1446. https://doi.org/10.1111/nph.16249
Shivaprakash, S.H., and S.E. Burns. 2025. Microbial interactions during carbonate biomineralization via urea hydrolysis metabolic pathway. *Acta Geotechnica* 20: 3929–3950. https://doi.org/10.1007/s11440-025-02609-8
Stubbendieck, R.M., D.S. May, M.G. Chevrette, M.I. Temkin, E. Wendt-Pienkowski, J. Cagnazzo, C.M. Carlson, J.E. Gern, et al. 2019. Competition among Nasal Bacteria Suggests a Role for Siderophore-Mediated Interactions in Shaping the Human Nasal Microbiota. *Applied and Environmental Microbiology* 85: e02406-18. https://doi.org/10.1128/AEM.02406-18
Swanner, E.D., A.M. Mloszewska, O.A. Cirpka, R. Schoenberg, K.O. Konhauser, and A.

Kappler. 2015. Modulation of oxygen production in Archaean oceans by episodes of Fe(II) toxicity. *Nature Geoscience* 8: 126–130. https://doi.org/10.1038/ngeo2327

UNEP. 2023. Global Framework on Chemicals – For a Planet Free of Harm from Chemicals and Waste. United Nations Environment Programme, Bonn, Germany. 21 pp. https://www.unep.org/resources/global-framework-chemicals-planet-free-harm-chemicals-and-waste

Uwimpaye, F., G. Twagirayezu, I.O. Agbamu, K. Mazurkiewicz, and J. Jeż-Walkowiak. 2025. Riverbank filtration: a frontline treatment method for surface and groundwater—African perspective. *Environmental Monitoring and Assessment* 197: 160. https://doi.org/10.1007/s10661-024-13413-4

Wang, Y., R. Branicky, A. Noë, and S. Hekimi. 2018. Superoxide dismutases: Dual roles in controlling ROS damage and regulating ROS signaling. *Journal of Cell Biology* 217: 1915–1928. https://doi.org/10.1083/jcb.201708007

Zhao, Y., A. Bitzer, J.J. Power, D. Belikova, B.O. Torres Salazar, L.A. Adolf, D. Gerlach, B. Krismer, et al. 2024. Nasal commensals reduce Staphylococcus aureus proliferation by restricting siderophore availability. *The ISME Journal* 18: wrae123. https://doi.org/10.1093/ismejo/wrae123
